# QTL mapping and underlying genes for heat tolerance in grapevine (Rhine Riesling × Cabernet Sauvignon) under field conditions

**DOI:** 10.1007/s00122-025-04972-2

**Published:** 2025-07-21

**Authors:** Silvia Pettenuzzo, Luca Cappellin, Michele Faralli, Maria Stella Grando, Laura Costantini

**Affiliations:** 1 Center Agriculture Food and Environment (C3A), University of Trento, San Michele All’Adige, Italy; 2 Research and Innovation Centre, Fondazione Edmund Mach, San Michele All’Adige, Italy; 3 Department of Chemical Sciences, University of Padova, Padua, Italy; 4 Department of Civil, Environmental and Architectural Engineering, University of Padova, Padua, Italy

## Abstract

**Key message:**

QTL analysis for key physiological traits assessed during hot days highlighted 26 genomic regions and promising candidate genes for thermotolerance and response to light stress under field conditions in grapevine.

**Abstract:**

Grapevine is one of the most widely cultivated perennial fruit crops in the world, with its economic relevance mainly related to wine production. Climate change, with global warming and increased frequency of intense phenomena, is greatly affecting viticulture and the wine sector. Thus, studying the genetic factors involved in grapevine response to high temperatures can help to improve vineyard management strategies and support plant breeding innovations. In this experiment, a mapping population (Rhine Riesling × Cabernet Sauvignon) was used to perform a genetic dissection of the physiological response to increased temperatures under vineyard conditions. Photosynthetic activity and stomatal dynamics were evaluated for three seasons during hot days at different plant developmental stages. Results of quantitative trait loci (QTL) analysis highlighted 26 genomic regions that consistently contribute to the eight tested traits. Candidate genes with supporting evidence, underlying QTL clusters with explained variance above 10%, are those associated with signal perception and transduction, protein homeostasis, osmoprotection, photosynthesis and response to radiation which are relevant mechanisms for plant heat acclimation. Within the stable chromosomal intervals identified by this exploratory analysis, other gene predictions emerged that may be tested for their involvement in grapevine resilience to increasing temperatures. The genetic architecture of quantitative traits linked to grapevine heat tolerance investigated under real field conditions, helps to define key targets for adapting an important traditional crop to environmental changes.

**Supplementary Information:**

The online version contains supplementary material available at 10.1007/s00122-025-04972-2.

## Introduction

Nowadays global warming has become an issue because of the increased frequency of intense phenomena (e.g., heat waves, heavy rains) that have adverse impacts on both ecosystems and human systems (IPCC 2023). One of the worst affected sectors is agriculture and consequently the food production chain. In fact, increased temperatures, often coupled with periods of water deficit, reduce yield and food quality with negative consequences on an economic level. Viticulture, with its 258 mhl of wine production and 37.6 bn EUR of global trade value (OIV (International Organisation of Vine and Wine) 2023), is one of the most relevant agricultural sectors at an economic level, and is highly impacted by climate change. Indeed, increased temperatures, compromise yield and influence phenological timings, anticipating berry ripening in the summer heat, leading for example to a higher alcohol content and lower acidity in wines with consequent modification of their sensory profile (Jones and Davis 2000; Van Leeuwen et al. 2019; Faralli et al. 2024). A future warmer climate will threaten the sustainability of viticulture in the current winemaking regions, especially in southern Europe, where temperatures are expected to increase above optimum values, accompanied by severe dryness (Santos et al. 2020). With the aim of improving the management of existing varieties and developing new cultivars with enhanced performances under changing environmental conditions, the study of the genetic factors involved in grapevine response to increased temperatures is essential to gain knowledge about the mechanisms underlying thermotolerance (Fahad et al. 2017). In this framework, quantitative trait loci (QTL) and genome-wide association studies (GWAS) are of utmost importance for an initial analysis of the complex phenotype, combining character investigation with genetic dissection. QTL mapping and GWAS have been successfully applied in the study of biotic stresses, with the discovery of many resistance loci for pests and diseases, also in grapevine (Delrot et al. 2020). Regarding the genetic control of abiotic stress tolerance, drought stress has received considerable attention and has been extensively studied in the past years in major annual crops, but also in walnut (Arab et al. 2022), apple (Wang et al. 2018) and grapevine (Marguerit et al. 2012; Coupel-Ledru et al. 2014, 2016, 2022; Brault et al. 2021; Trenti et al. 2021), which led to the discovery of various QTL, meta-QTL and candidate genes related to yield, root systems and stomatal conductance traits. In recent years, crop species have been intensively studied for thermotolerance too, with first QTL analysis on wheat (e.g., Paliwal et al. 2012) followed by tomato (e.g., Cappetta et al. 2021), maize (e.g., Sheoran et al. 2022) and rice (e.g.. Kilasi et al. 2018), sorghum (Chen et al. 2017), groundnut (Sharma et al. 2023), chickpea (Mohanty et al. 2024) and cowpea (Angira et al. 2022), while literature on perennial crops is very poor. All these works described a polygenic control for heat-responsive traits. Only a few quantitative genetics investigations are reported for the grapevine response to changes in temperature, including a QTL analysis on cold hardiness (Su et al. 2020) and two GWAS on cold tolerance (Wang et al. 2021b) and leaf firing (Coupel-Ledru et al. 2024). Therefore, the genetic architecture of the grapevine response to rising temperatures remains to be explored.

In thermotolerance studies the most frequently considered trait is plant photosynthetic activity. Indeed, together with genetic studies, indicators of photosynthetic performance were used to investigate varietal behavior in heat stress studies of several crop species, such as wheat (Monneveux et al. 2003; Sharma et al. 2012, 2015), maize (Sinsawat et al. 2004), raspberry (Molina-Bravo et al. 2011), tropical fruits (Yamada et al. 1996), tomato (Willits and Peet 2001; Zhou et al. 2015; Poudyal et al. 2019) and particularly in grapevine (Kadir 2006; Palliotti et al. 2009; Greer and Weston 2010; Luo et al. 2011; Greer and Weedon 2012; Xu et al. 2014; Hochberg et al. 2015; Sun et al. 2016, 2017; Xiao et al. 2016; Zha et al. 2016b, a, 2018; Greer 2017, 2019; Liu et al. 2020b; Gallo et al. 2021). Actually, photosynthesis is the first plant process influenced by thermal variation: high temperatures can cause an increase in thylakoid membranes fluidity, which favors the disruption of the oxygen evolving complex (OEC), and can reduce the activity of Rubisco (ribulose-1,5-bisphosphate carboxylase/oxygenase), impairing the chloroplasts major biochemical processes (Wahid et al. 2007; Fahad et al. 2017; Venios et al. 2020).

Several methods can be used for assessing key-steps of processes associated with leaf photosynthesis. Among them, the activity of Photosystem II (PSII) can be estimated via chlorophyll fluorescence emission in dark-adapted samples. Light absorbed by chlorophyll, in fact, can be employed for photosynthesis (photochemistry) or can be either dissipated through heat (non-photochemical quenching, NPQ) or re-emitted as light (fluorescence) (Murchie and Lawson 2013). Therefore, the light emitted from the Photosystem II (PSII) in the absence of NPQ can give information on the status of the photochemical process, providing a good indication of plant status. This technique is particularly attractive because it is non-invasive and non-destructive for plants, and measurements are simple and relatively fast. Moreover, the quantification of the light re-emitted by chlorophyll is usually done with portable instruments (fluorimeters) that can be easily used in field experiments (Maxwell and Johnson 2000).

A reduced leaf transpiration due to stomata closure to minimize water loss results in an increase in leaf temperature under high light conditions, which, combined with heat stress phenomena, can increase leaf sensitivity to sub-optimal environmental conditions. Since water conservation via high stomatal sensitivity to environmental cues could be relevant in the scenario of climate change (Faralli et al. 2019), investigating plant stomatal behavior holds significant importance. However, studies performing QTL analysis on stomatal dynamics in relation to abiotic stress factors are very few in literature and mainly related to salinity stress (Liu et al. 2017) or drought stress (Wang et al. 2003; Trenti et al. 2021).

In the present work, a biparental population obtained from the cross between Rhine Riesling, a German variety adapted to cool-moderate temperatures, and Cabernet Sauvignon,’ one of the world most widespread varieties cultivated in hot-temperate climates, was used to perform a genetic dissection of grapevine physiological response to increased temperatures under field conditions. Photosynthetic activity and stomatal dynamics were evaluated for three consecutive seasons during hot days at different plant developmental stages, in particular flowering and véraison, which are known to be sensitive to temperature increases (Yeh et al. 2012; Jagadish et al. 2021). QTL analysis for these traits highlighted several QTL stable across years, phenotyping sessions or parameters. The investigation of these genomic regions allowed the identification of genes putatively involved in the response to heat stress or solar radiation, contributing to a deeper understanding of the mechanisms involved in grapevine thermotolerance.

## Materials and methods

### Plant materials

The study was conducted for 3 years (2021–2023) on 122 grapevine individuals obtained from the cross ‘Rhine Riesling’ × ‘Cabernet Sauvignon’ (RRxCS). The cross was made in 2005 and the seedlings were planted in the field on their own roots in 2008. Plants were grown at the Giaroni experimental field of Fondazione Edmund Mach (San Michele All’Adige, Trentino Alto Adige, Italy, 46°18′N, 11°13′E) and were trained using the Guyot system. Analysis of the soil texture showed that Giaroni soil is typically a sandy loam soil (the percentage of sand was 52.5% with 41.9% silt and 5.6% clay, < 1% skeleton) with a soil depth of 0.70–1 m and an estimated available water content (AWC) of 164 mm. During the growing season plants were regularly treated against common grapevine pathogens and underwent two seasonal trimmings. Weather conditions during the study were monitored with an automated meteorological station situated near the vineyard (https://meteo.fmach.it, Fondazione Edmund Mach, Technology Transfer Center, Agrometeorology and Irrigation Unit).

### Phenotypic characterization

Chlorophyll a fluorescence transient (O-J-I-P) was measured with a Handy Plant Efficiency Analyzer (Handy-PEA, Hansatech, Norfolk, UK) set up with a light intensity of the saturating pulse of 3500 µmol m^−2^ s^−1^. For parental lines, 5 biological replicates were studied, while 1 biological replicate was available for the progeny. For each plant, three measures were taken on the 5th leaf of three different shoots starting from the shoot base. Shoots were chosen from the head, middle, and end of the cane. Before measurement each leaf was dark-adapted for 30 min with suitable leaf clips. The same leaves were measured throughout the summer season.

In 2022 and 2023, stomatal conductance (*g*_s_) and leaf vapor pressure (*VPleaf*), leaf temperature (*Tleaf*), light-adapted maximum quantum yield of the Photosystem II (*PhiPS2*), electron transport rate (*ETR*) and leaf apparent transpiration (*E*) were measured with a LI-600 Porometer/Fluorometer (LI-COR Environmental, Lincoln, Nebraska USA). “Auto gsw” was selected as protocol for LI-COR measurements.

Measures in non-stress conditions (control, C) were taken in the early morning (5:30–8:00), while measures in stress conditions (heat stress, HS) were collected in the afternoon during the hottest hours (13:00–15:30). To ensure the proper temperature and light conditions during control measurements, the population was divided into two subgroups and evaluated on two consecutive hot days. Measures in 2021 were taken at flowering (E-L21, beginning of June) and véraison (E-L35, beginning of August). In 2022 and 2023, in addition to these stages, two other datasets were collected at the end of June and in July (corresponding to berry pea-size, E-L31, and pre-véraison E-L33, respectively), while in 2023 an additional dataset was acquired at full véraison in August (E-L36), when the highest air temperature was reached.

### QTL analysis

The RRxCS population was genotyped with an Illumina Vitis18K SNP chip and SSR markers in a previous work and a high-density linkage map was obtained (Vervalle et al. 2022). The integrated map contained 3459 representative markers mapped at 1815 unique positions along 19 linkage groups, with a length of 1413 centiMorgan (cM) and an average inter-locus gap of 0.78 cM. For each individual of the RRxCS progeny and for each phenotyping session the difference between stress and non-stress conditions of measured parameters was normalized for control values (formula: (C-HS) * C^−1^ * 100). According to the literature (Talukder et al. 2014) and a preliminary QTL analysis, this approach was considered more robust than analyzing datasets obtained from simple subtraction between data at control and during stress. QTL detection was carried out on the integrated map (named ‘CP map’, from cross-population) and the two parental maps (named ‘RR map’, from Rhine Riesling, and ‘CS map’, from Cabernet Sauvignon) using the MapQTL 6.0 software (Van Ooijen 2009) with data from separate phenotyping sessions. Simple interval mapping (SIM) was performed for all three maps with the mixture model algorithm, while multiple QTL mapping (MQM) was performed only on the two parental maps due to its high computation cost. MQM analysis was based on the Forward Selection approach because it appeared less prone to false positives compared with Automatic Cofactor Selection. LOD (logarithm of odds) significance thresholds at a P value of 0.05 were established for each linkage group through 1000 permutations. A nonparametric Kruskal–Wallis test was carried out to provide support to marker-trait associations. The QTL confidence interval (in cM) was calculated as the genetic region where the LOD value was higher than the maximum LOD score of the QTL minus 1. For confirmed QTL in at least two datasets, potential candidate genes were investigated in the consensus region (i.e., QTL overlapping region or, in the case of two adjacent QTL, the entire region was considered). Markers’ physical positions (in base pair, bp) were obtained by blasting the probe sequences (for SNPs) or the repetitive sequences (for SSRs) on the PN40024.v4 reference (REF) genome assembly. For PN40024.v4 heterozygous regions, alternative (ALT) sequences were also considered (Velt et al. 2023). Structural and functional gene annotations (in the v4.3 and v4.1 version, respectively) were recovered from the Grape Genomics Encyclopedia portal (https://grapedia.org/) (Velt et al. 2023). Candidate genes were selected, among those included in the QTL confidence intervals, based on the knowledge of the main mechanisms that plants have evolved to counteract the effects of heat stress as well as on previous works exploring the transcriptomic response of grapevine to heat stress.

Among the traits measured and calculated by the Handy-PEA fluorometer in relation to chlorophyll fluorescence (Table S 1.1, Online Resource 1), in this study four parameters were selected for QTL analysis: *F*_*v*_*/F*_*m*_, which gives an estimation of the maximum quantum efficiency of PSII; *Area*, which is proportional to the pool size of the electron acceptors *Q*_A_^−^ on the reducing side of PSII; *Phi(Eo)*, which is the quantum yield of electron transport; and *Phi(Ro)*, which is the average quantum yield of primary photochemistry. These independent parameters were chosen based on their physiological meaning. In fact, many parameters reported in Table S 1.1 are calculated and derived from others and therefore they are expected to display the same trend and results in the QTL analysis.

Traits related to stomatal dynamics and selected for QTL analysis were: *g*_s_, which measures stomatal conductance in vivo; *E,* which gives an estimation of leaf transpiration; *VPleaf,* which measures leaf vapor pressure; *Tleaf,* which corresponds to leaf temperature; *PhiPS2*, which gives an estimation of the maximum quantum efficiency of PSII without dark adaptation; and *ETR*, which gives an estimation of the electron transport rate (complete list of measured parameters in Table S 1.2, Online Resource 1).

### Statistical analysis and graphical representation

Statistical analysis and graphical representation were performed using the R software environment 4.2.1 (https://www.r-project.org/). The normality of each trait distribution was evaluated by the Shapiro–Wilk Normality Test. Correlations between physiological traits measured in different phenotyping sessions and years were calculated with the package ‘tidyverse’ using the nonparametric Spearman correlation coefficient (rho). QTL intervals were visualized in a single image with the R package ‘RIdeogram.’

## Results

### Environmental conditions

**Fig. 1:**
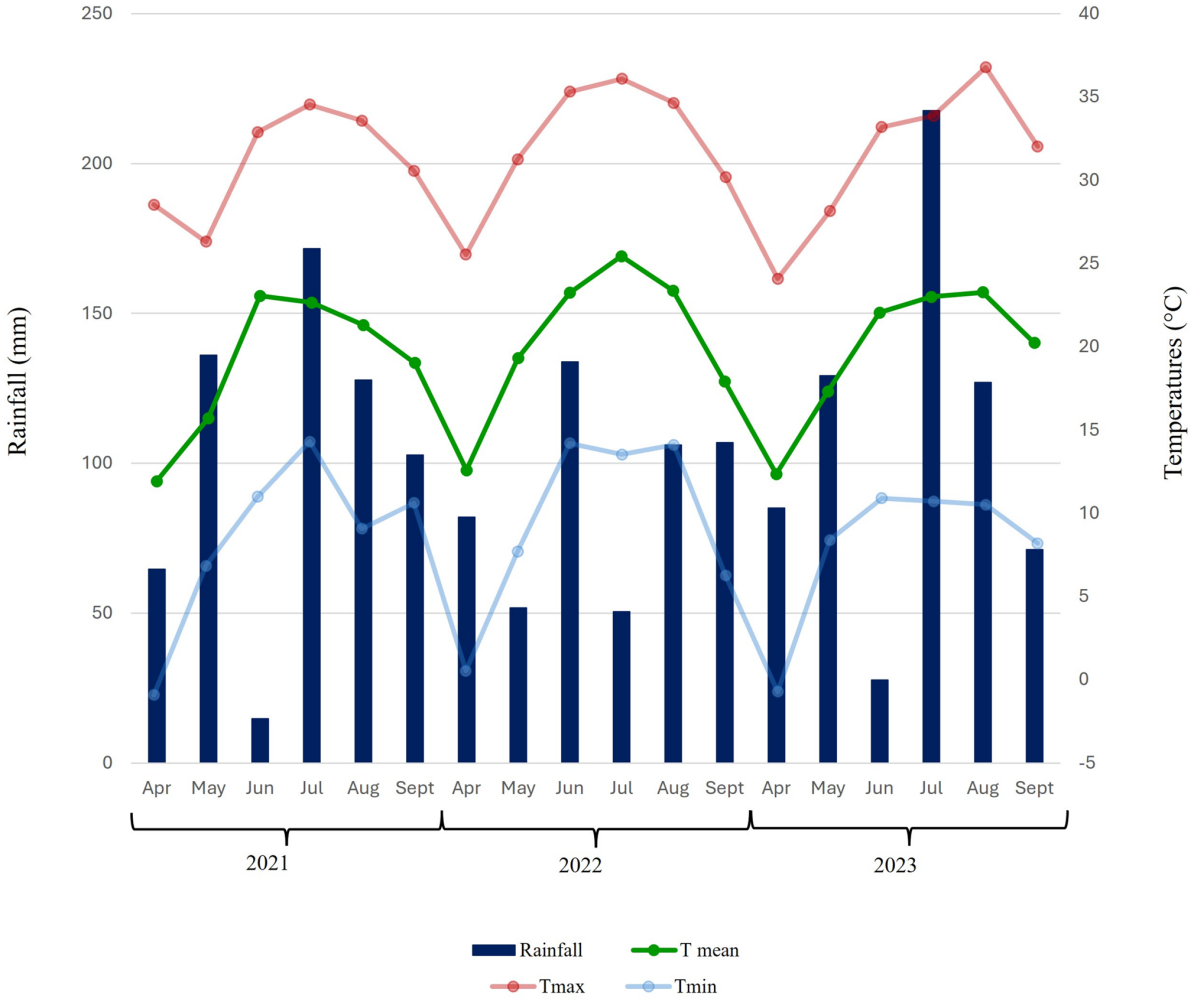
Climograph reporting precipitations (mm) and air temperatures (mean in green, maximum in red and minimum in light-blue) in Giaroni experimental field (Trentino Alto Adige, Italy, 46°18′N, 11°13′E), for seasons 2021–2023 in the period between bud burst-harvest (April–September) (colour figure online)

The period from bud burst to harvest (April-September) was characterized by moderate temperatures and approximately ~ 600 mm of cumulative precipitations (Fig. 1) well distributed over the growing season. Due to the near-hydromorphic nature of the soil located close to the Adige River and the maximum water-holding capacity reached at the beginning of each season, no significant level of soil moisture deficit occurred during the three vintages. Years 2022 and 2023 were characterized by mean temperatures, both in their minimum and maximum values, about ~ 1 °C higher compared to 2021, with 2022 having the highest maximum mean temperature of 27.8 °C, and a maximum air temperature of 36.6 °C for two days in July. In 2023, the maximum air temperature recorded during the study, 36.8 °C, was reached in August (Fig. 1).

Phenotyping for QTL analysis related to heat-responsive traits was performed at different phenological stages. Detailed temperature information for all the phenotyping sessions is reported in Table 1. In 2021 and 2023, temperatures during flowering phenotyping sessions were low; therefore, those datasets were not included in the present study.

**Table 1:** For each phenotyping session performed, dataset code, phenotyping date, day mean (Day mean T), maximum (Air *T*_max_) and minimum (Air *T*_min_) air temperature, temperature difference between morning and afternoon phenotyping sessions (Δ*T*) and monthly total precipitation are reported

Dataset code	Phenotyping dates	Day mean *T* (°C)	Air *T*_max_ (°C)	Air *T*_min_ (°C)	Δ*T* (°C)	Monthly total rainfall (mm)
Véraison 2021	11/08/2021	24.0	32.3	19.0	13.3	127.8
	12/08/2021	24.9	33.0	18.5	14.5	
	Mean	24.5	32.7	18.8	13.9	
Flowering 2022	02/06/2022	22.4	28.8	15.3	13.5	133.8
	04/06/2022	24.0	30.3	18.7	11.6	
	Mean	23.2	29.6	17.0	12.6	
Berry pea-size 2022	20/06/2022	24.0	32.0	15.8	16.2	133.8
	21/06/2022	23.9	31.1	18.5	12.6	
	Mean	24.0	31.6	17.2	14.4	
Pre-Véraison 2022	19/07/2022	27.3	34.8	17.5	17.3	50.4
	20/07/2022	27.7	35.9	17.4	18.5	
	Mean	27.5	35.4	17.5	17.9	
Véraison 2022	03/08/2022	25.0	32.8	17.5	15.3	106
	05/08/2022	27.0	35.0	18.5	16.5	
	Mean	26.0	33.9	18.0	15.9	
Berry pea-size 2023	20/06/2023	25.1	30.5	19.0	11.5	27.8
	21/06/2023	25.1	31.7	18.2	13.5	
	Mean	25.1	31.1	18.6	12.5	
Pre-Véraison 2023	11/07/2023	28.2	34.2	21.8	12.4	217.6
	17/07/2023	27.3	33.4	21.6	11.8	
	Mean	27.7	33.8	21.7	12.1	
Véraison 2023	09/08/2023	19.2	25.7	14.7	11.0	127
	10/08/2023	22.7	28.9	17.0	11.9	
	Mean	20.9	27.3	15.9	11.5	
Véraison Full 2023	22/08/2023	**27.4**	**36.8**	**20.4**	**16.4**	127

Datasets with the highest average maximum air temperature were *Pre-Véraison 2022* and *Véraison Full 2023*, with values of 35.4 °C and 36.8 °C, respectively. The corresponding average ΔT values were 17.9 and 16.4 °C. For the other collected datasets, the average maximum air temperature ranged from 27.3 to 33.9 °C and the average ΔT ranged between 11.5 and 15.9 °C.

### Phenotypic characterization

**Fig. 2:**
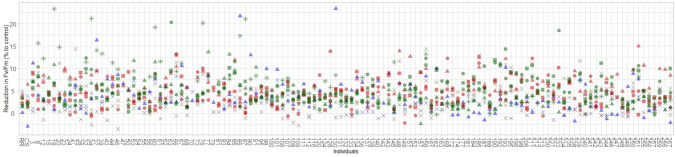
Reduction in *F*_*v*_/*F*_*m*_ (% relative to control) in different phenotyping sessions for all the individuals of the mapping population (‘Rhine Riesling’ × ’Cabernet Sauvignon’) reported with their numerical codes. Phenotyping sessions are indicated as follows: ▲ flowering, ■ berry pea-size, ● pre-véraison, × véraison, * véraison full; while dots are colored based on the year: 2021 (blue), 2022 (red), 2023 (green) (colour figure online)

Mean values and ranges of variation for parameters related to chlorophyll fluorescence analyzed in this study in all the datasets collected between 2021 and 2023 are reported in Table S 1.3 (Online Resource 1). For all parameters, there was a decrease in the afternoon compared to control values. The four parameters considered in the study (*F*_*v*_*/F*_*m*_*, Area, Phi(Ro), Phi(Eo)*) were, in general, positively correlated within all datasets collected, with rho values ranging from 0.21 (*F*_*v*_*/F*_*m*_ and *Phi(Ro)* in *Berry pea-size 2023*) to 0.90 (*F*_*v*_*/F*_*m*_ and *Phi(Eo)* in *Pre-Véraison 2023*) (Table S 1.4, Online Resource 1). On the contrary, correlations registered for the same parameter across different phenotyping sessions were in general small and often not significant, especially when comparing datasets belonging to different years (Table S 1.5, Online Resource 1). For example, for the *F*_*v*_*/F*_*m*_ parameter, it is evident that individuals’ behavior changed not only between different years, but also along the season (Fig. 2).

Significant correlations of *F*_*v*_*/F*_*m*_ across years ranged between 0.21 and 0.36, while correlations ranged between 0.21 and 0.73 when considering datasets from the same year (Table S 1.5- a, Online Resource 1). Parental lines showed a more consistent behavior throughout different years and seasons also because the reduction in *F*_*v*_*/F*_*m*_ reported here is the mean of 5 biological replicates. Frequency distribution plots of the difference between stress and non-stress conditions of measured parameters (normalized for control values, formula: (C-HS) * C^−1^ * 100) were approximately normal and showed continuous variation (Figure S 1.1, Online Resource 1). Rhine Riesling and Cabernet Sauvignon displayed a similar phenotype and transgressive segregants among the population were observed.

Regarding transpirational traits, we observed a general trend of increasing stomatal conductance and leaf transpiration in the afternoon (Table S 1.6, Online Resource 1). This trend was also observed for both parental lines, Rhine Riesling and Cabernet Sauvignon (data not shown). High correlations (0.93–0.999) were registered for *Tleaf* and *VPleaf* in all datasets acquired, while *g*_s_ and *E* in general did not correlate with *Tleaf* (Table S 1.7, Online Resource 1). All stomatal and transpirational parameters negatively correlated with *PhiPS2*, but the degree of correlation varied with the dataset considered. As for parameters measured with Handy-PEA, correlations of the same parameter between different datasets were higher when considering datasets from the same year (Table S 1.8, Online Resource 1). Correlations between traits measured with Handy-PEA and LI-600 porometer/fluorometer in the same phenotyping session were additionally calculated (Table S 1.9, Online Resource 1). In this case, correlations depended on the considered dataset, making it difficult to highlight common trends between datasets. However, there were significant and positive correlations between *F*_*v*_*/F*_*m*_ and *PhiPS2*, with rho values ranging between 0.28 and 0.52, while *Tleaf* negatively correlated with *Area*, *F*_*v*_*/F*_*m*_ and in some cases also with *Phi(Eo)*. Frequency distribution plots for parameters measured with LI-600 Porometer/Fluorometer are available in Figure S 1.2, Online Resource 1. These traits appear to have a non-normal distribution; the progeny tends to display values which are similar to the parental lines, but transgressive segregation can still be observed.

### QTL analysis

#### Chlorophyll fluorescence

**Fig. 3:**
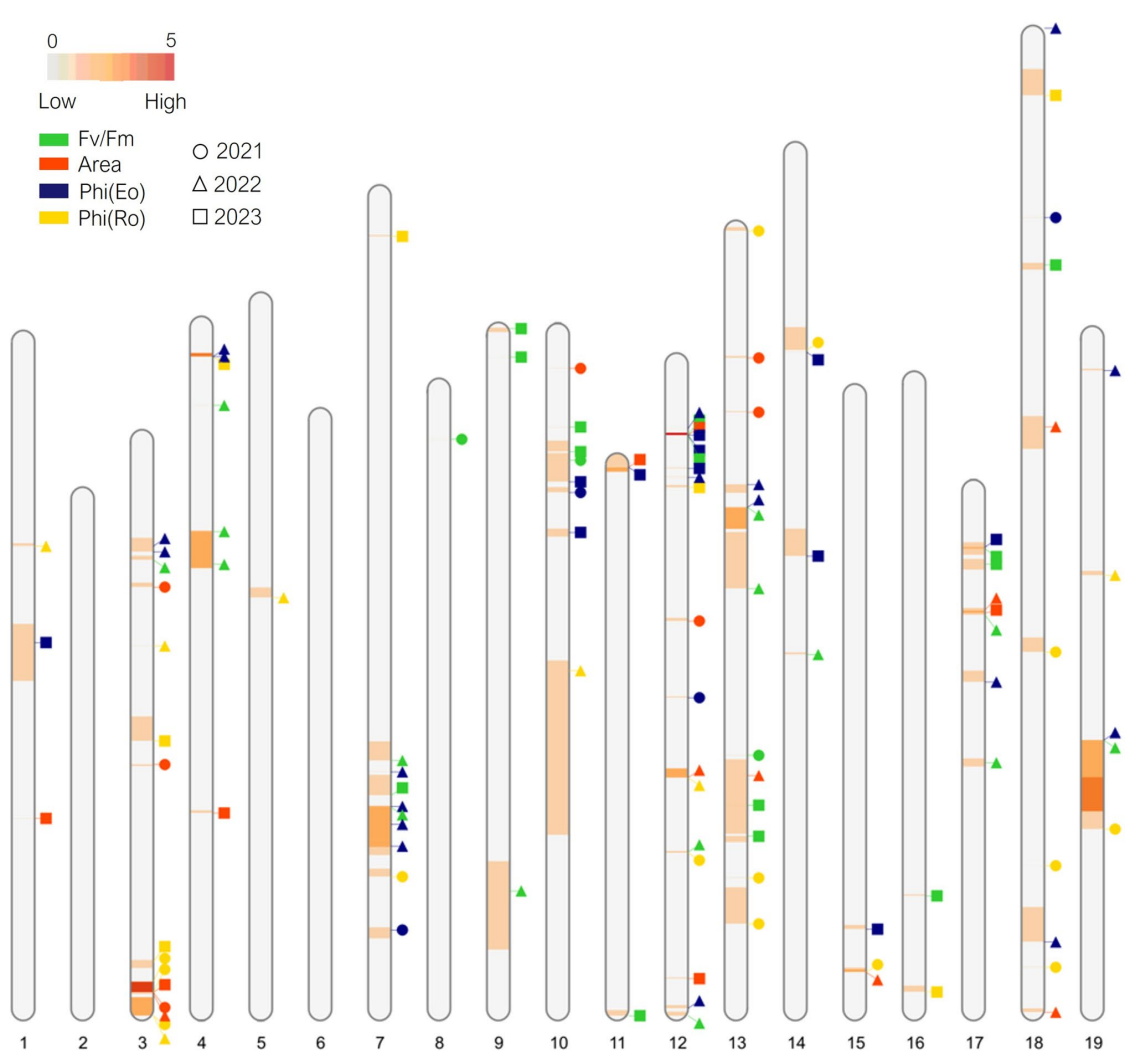
Results of MQM analysis (2021–2023) on chlorophyll fluorescence parameters. Parameters are represented with different colors (*F*_*v*_/*F*_*m*_ in green, *Area* in red, *Phi*(*Eo*) in blue and *Phi*(*Ro*) in yellow) while years are reported with different shapes (● 2021, ▲ 2022, ■ 2023). Chromosomes are colored following the increasing frequency with which a genomic region harbors significant QTL according to MQM analysis: from 0 (gray) to 5 repetitions (red) across different datasets and/or parameters. The size of genomic regions highlighted in the figure is reported in basepairs (colour figure online)

SIM analysis performed on the integrated map with data from a total of 9 phenotyping sessions over three years resulted in the identification of 56 QTL: 19 for the maximum quantum yield of Photosystem II (*F*_*v*_*/F*_*m*_), 10 for the pool size of electron transport (*Area*), 15 for the quantum yield of electron transport (*Phi(Eo)*), and 12 for the average quantum yield of primary photochemistry (*Phi(Ro)*) (Table S 2.1, Online Resource 2). QTL identified in the integrated map have a LOD in the range 1.9–5.5 and a phenotypic explained variance (EV%) between 7.6 and 21.5%. On the other hand, MQM analysis performed on parental maps highlighted 96 QTL: 27 for *F*_*v*_*/F*_*m*_, 17 for *Area*, 30 for *Phi(Eo)*, and 22 for *Phi(Ro)*, respectively (Table S2.2, Online Resource 2). QTL identified in parental maps have a LOD in the range 1.6–5.4 and their EV is between 4 and 17.4%. QTL related to chlorophyll fluorescence appear to be distributed in almost all 19 chromosomes (Chr) of the grapevine genome, with Chr 3, 4, 7, 10, 12, 13, 17, and 19 being the most populated (see Figure S 2.1 in Online Resources 2 as a summary of QTL obtained from SIM analysis on the integrated map and MQM analysis on parental maps). As shown in Fig. 3, some of the QTL obtained from the MQM analysis co-locate, highlighting genomic regions worthy of investigation for candidate genes.

Oppositely, SIM and MQM analysis performed with data from a total of 5 datasets for chlorophyll fluorescence parameters measured with the LI-600 porometer, *PhiPS2* and *ETR*, did not highlight genomic regions repeated across phenotyping sessions or years (data not shown). Moreover, no common regions were found in QTL analyses performed for *F*_*v*_*/F*_*m*_ and *PhiPS2*, parameters measured with (Handy-PEA) and without (LI-600 Porometer/Fluorometer) leaf dark adaptation, respectively. Therefore, those regions were not further investigated for candidate genes.

#### Stomatal conductance

SIM analysis performed on the integrated map with data from a total of 5 different datasets collected in 2 years highlighted 26 QTL: 5 related to stomatal conductance (*g*_s_), 6 related to leaf transpiration (*E*), 7 for leaf vapor pressure (*VPleaf*), and 8 for leaf temperature (*Tleaf*) (Table S 2.3 Online Resource 2). QTL identified in the integrated map have a LOD in the range 1.5–6.1 and a phenotypic explained variance between 6 and 22.2%. On the other hand, MQM analysis performed on parental maps resulted in the identification of 41 QTL: 14 for *g*_s_, 14 for *E*, 5 for *VPleaf*, 8 for *Tleaf* (Table S 2.4, Online Resource 2). QTL identified in parental maps have a LOD in the range 0.7–3.8 and their EV is between 0.1 and 12.5%.

**Fig. 4:**
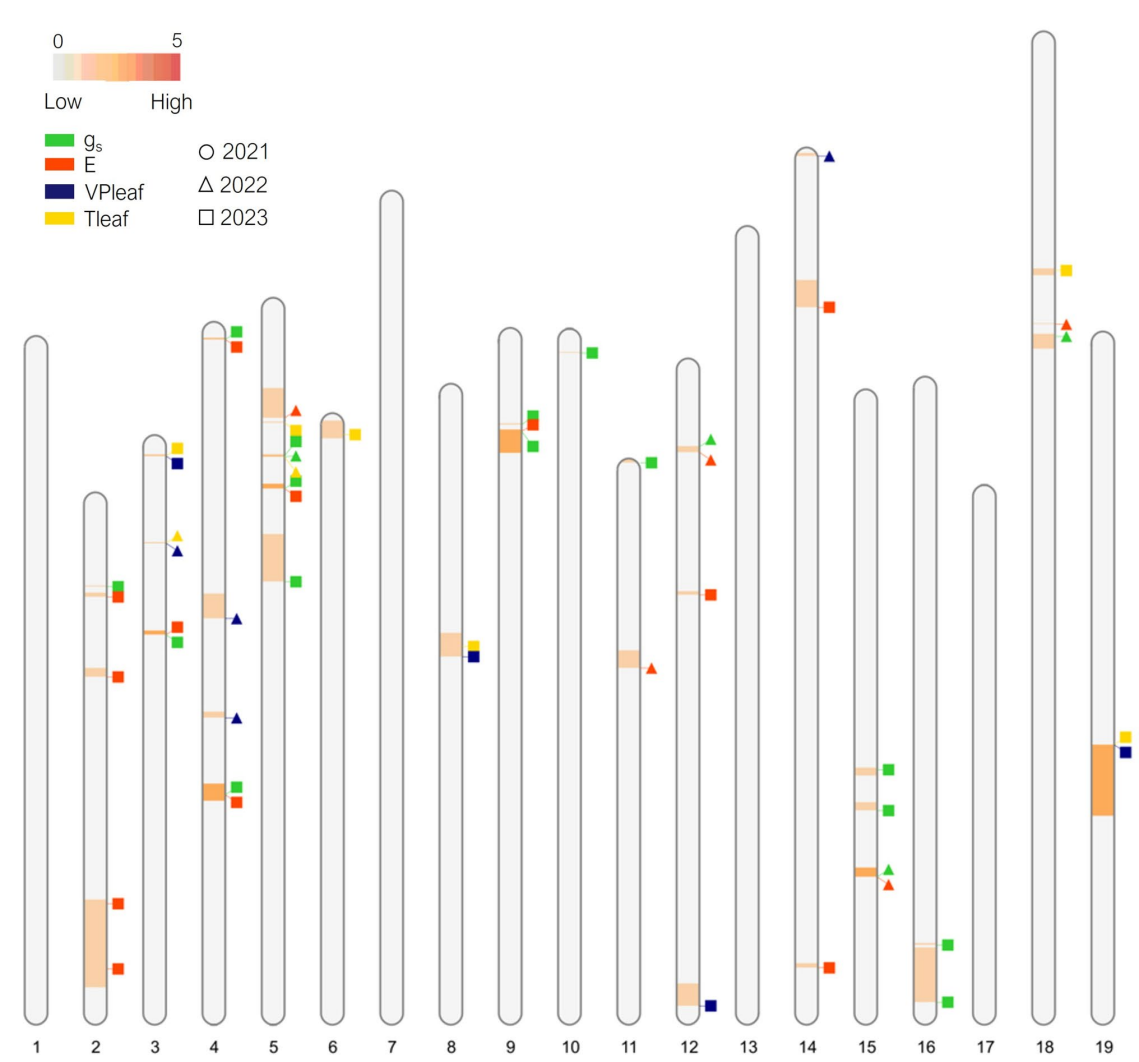
Results of MQM analysis (2021–2023) on leaf transpirational parameters. Parameters are represented with different colors (*g*_s_ in green, *E* in red, *VPleaf* in blue and *Tleaf,* in yellow) while years are highlighted with different shapes (● 2021, ▲ 2022, ■ 2023). Chromosomes are colored following the increasing frequency with which a genomic region harbors significant QTL according to MQM analysis: from 0 (gray) to 5 repetitions (red) across different datasets and/or parameters. The size of genomic regions highlighted in the figure is reported in basepairs (colour figure online)

QTL related to stomatal conductance and leaf transpiration are fewer in number and less distributed across chromosomes compared to QTL for chlorophyll fluorescence, with Chr 3 and 5 being the most populated (see Figure S 2.2 in Online Resources 2 as a summary of QTL obtained from SIM analysis on the integrated map and MQM analysis on parental maps). Despite the frequency of their repetition throughout different datasets being quite low, 10 genomic regions with co-localized QTL and underlying candidate genes were identified (Fig. 4).

Consensus genomic regions, with stable QTL identified throughout years and/or phenological stages, were considered for candidate genes exploration (Tables 2, 3). Functional candidate genes that have been associated with the transcriptomic response of plants to heat stress, are summarized in Table 4. The complete list of positional candidate genes within each region is reported in Table S 3.1 and Table S 3.2, Online Resource 3, for QTL related to chlorophyll fluorescence and stomatal dynamics, respectively.

**Table 2:** Consensus genomic regions (indicated with a QTL code and reported in base pairs on the reference genome assembly PN40024.v4) in which QTL for chlorophyll fluorescence (cf) kinetics related to heat stress co-locate. The number of QTL detected, the genomic regions corresponding to their confidence intervals, the associated traits and datasets, the explained variance (EV%) and the number of genes in the interval are also indicated. QTL clusters originated by QTL with EV > 10% are highlighted in bold

Consensus genomic region				QTL co-locating						n genes
QTL code	Chr	Start (bp)	End (bp)	n QTL	Trait	Dataset	Start (bp)	End (bp)	EV%	
cfQTL_3.1*	3	19,392,699	19,751,147	4	*Area*	Véraison 2021	19,392,699	19,751,147	14.2	19
					*Area*	Flowering 2022	19,392,699	19,813,686	9.4	
					*Area*	Véraison Full 2023	19,392,699	19,751,147	8.1	
					*Phi(Ro)*	Véraison 2022	19,363,439	19,392,699	16.3	
*cfQTL_3.2*	3	19,929,243	20,575,870	2	*Phi(Ro)*	Véraison 2021	19,929,243	20,575,870	7.2	43
					*Phi(Ro)*	Flowering 2022	19,929,243	20,575,870	9.5	
***cfQTL_4.1***	4	1,295,066	1,419,855	3	*Phi(Eo)*	Berry pea-size 2022	1,295,066	1,419,855	11.6	14
					*Phi(Eo)*	Pre-Véraison 2022	1,295,066	1,419,855	12.2	
					*Phi(Ro)*	Véraison Full 2023	1,295,066	1,419,855	8.3	
*cfQTL_4.2*	4	7,561,727	8,842,722	2	*F*_*v*_*/F*_*m*_	Pre-Véraison 2022	7,526,331	8,842,722	8.1	56
					*F*_*v*_*/F*_*m*_	Véraison 2022	7,561,727	8,842,722	8.3	
***cfQTL_7.1***	7	21,817,724	23,247,875	2	*F*_*v*_*/F*_*m*_	Pre-Véraison 2022	21,817,724	23,247,875	8.5	147
					*Phi(Eo)*	Pre-Véraison 2022	21,805,347	21,817,724	10.8	
					*Phi(Eo)*	Berry pea-size 2022	21,817,724	22,449,811	7.1	
					*Phi(Eo)*	Véraison 2022	22,988,546	23,533,273	9	
cfQTL_10.1*	10	4,562,576	5,568,625	2	*F*_*v*_*/F*_*m*_	Véraison 2021	4,562,576	5,441,269	7	94
					*Phi(Eo)*	Véraison Full 2023	5,441,269	5,568,625	6.6	
*cfQTL_11.1*	11	493,591	664,729	2	*Phi(Eo)*	Véraison Full 2023	459,975	664,729	6.6	27
					*Area*	Véraison Full 2023	493,591	664,729	7.6	
***cfQTL_12.1***	12	2,814,814	2,886,340	5	*F*_*v*_*/F*_*m*_	Berry pea-size 2023	2,814,814	2,886,340	13.1	1
					*F*_*v*_*/F*_*m*_	Pre-Véraison 2023	2,814,814	2,886,340	8.3	
					*Area*	Berry pea-size 2023	2,814,814	2,886,340	8.2	
					*Phi(Eo)*	Berry pea-size 2023	2,814,814	2,886,340	9.1	
					*Phi(Eo)*	Véraison 2023	2,814,814	2,886,340	10.7	
*cfQTL_12.2*	12	14,585,617	14,910,568	2	*Area*	Véraison 2022	14,585,617	14,910,568	5.6	13
					*Phi(Ro)*	Berry pea-size 2022	14,585,617	14,910,568	5.5	
cfQTL_12.3*	12	17,505,010	17,528,465	2	*F*_*v*_*/F*_*m*_	Pre-Véraison 2022	17,505,010	17,528,465	6	2
					*Phi(Ro)*	Véraison 2021	17,505,010	17,542,846	6.7	
*cfQTL_13.1*	13	10,082,268	10,844,992	2	*F*_*v*_*/F*_*m*_	Flowering 2022	10,082,268	10,844,992	7	43
					*Phi(Eo)*	Flowering 2023	10,082,268	10,844,992	7	
*cfQTL_13.2*	13	20,532,927	20,532,927	2	*F*_*v*_*/F*_*m*_	Véraison Full 2023	20,532,927	20,532,927	6.5	126
					*Area*	Véraison 2022	18,926,455	21,543,135	6.2	
*cfQTL_15.1*	15	20,559,862	20,653,974	2	*Phi(Ro)*	Flowering 2022	20,559,862	20,653,974	7.5	22
					*Area*	Flowering 2022	20,498,981	20,653,974	7.5	
*cfQTL_17.1*	17	2,362,104	2,419,076	2	*Phi(Eo)*	Pre-Véraison 2023	2,362,104	2,629,802	7.1	39
					*F*_*v*_*/F*_*m*_	Berry pea-size 2023	2,203,470	2,419,076	9.8	
cfQTL_17.2*	17	4,591,415	4,678,056	2	*Area*	Berry-pea size 2023	4,591,415	4,678,056	9.8	22
					*F*_*v*_*/F*_*m*_	Véraison 2022	4,565,731	4,733,756	6.1	
***cfQTL_19.1***	19	15,845,364	17,044,447	3	*F*_*v*_*/F*_*m*_	Berry pea-size 2022	14,538,060	17,044,447	11.5	87
					*Phi(Eo)*	Berry pea-size 2022	14,538,060	17,044,447	15.9	
					*Phi(Ro)*	Véraison 2022	15,845,364	17,660,947	6.7

**Table 3:** Consensus genomic regions (indicated with a QTL code and reported in base pairs on the reference genome assembly PN40024.v4) in which QTL for stomatal dynamics (tr) related to heat stress co-locate. The number of QTL detected, the genomic regions corresponding to their confidence intervals, the associated traits and datasets, the explained variance (EV%) and the number of genes in the interval are also indicated. QTL clusters originated by QTL with EV > 10% are highlighted in bold

Consensus genomic region				QTL co-locating						n genes
QTL code	Chr	Start (bp)	End (bp)	n QTL	Trait	Dataset	Start (bp)	End (bp)	EV%	
*trQTL_3.1*	3	697,606	738,212	2	*Tleaf*	Pre-Véraison 2023	697,606	738,212	7.2	8
					*Vpleaf*	Pre-Véraison 2023	697,606	738,212	7	
***trQTL_3.2***	3	3,783,778	3,803,994	2	*Tleaf*	Flowering 2022	3,783,778	3,803,994	8.3	2
					*Vpleaf*	Flowering 2022	3,783,778	3,803,994	12	
*trQTL_3.3*	3	6,881,348	7,027,379		*g*_s_	Berry pea-size 2023	6,881,348	7,027,379	7.2	7
					*E*	Berry pea-size 2023	6,881,348	7,027,379	7.1	
*trQTL_4.1*	4	578,155	622,483	2	*g*_s_	Véraison 2023	578,155	622,483	0.1	8
					*E*	Véraison 2023	578,155	622,483	2.8	
*trQTL_4.2*	4	16,249,818	16,854,397	2	*g*_s_	Berry pea-size 2023	16,249,818	16,854,397	8.2	34
					*E*	Berry pea-size 2023	16,249,818	16,854,397	6.8	
***trQTL_5.1***	5	5,502,441	5,593,619	2	*g*_s_	Berry pea-size 2023	5,502,441	5,563,075	8.5	10
					*g*_s_	Berry pea-size 2022	5,563,075	5,593,619	12.5	
					*Tleaf*	Flowering 2022	5,563,075	5,593,619	8.1	
*trQTL_5.2*	5	6,546,178	6,717,310	2	*g*_s_	Véraison 2023	6,546,178	6,717,310	0.2	23
					*E*	Véraison 2023	6,546,178	6,717,310	3.7	
***trQTL_9.1***	9	3,585,046	4,406,275	2	*g*_s_	Berry pea-size 2023	3,585,046	4,406,275	10.3	99
					*E*	Berry pea-size 2023	3,585,046	4,406,275	10.1	
*trQTL_15.1*	15	16,825,206	17,149,995	2	*g*_s_	Berry pea-size 2022	16,825,206	17,149,995	5.8	26
					*E*	Berry pea-size 2022	16,825,206	17,149,995	5.5	
*trQTL_19.1*	19	14,538,060	17,044,447	2	*Tleaf*	Pre-Véraison 2023	14,538,060	17,044,447	8.6	65
					*Vpleaf*	Pre-Véraison 2023	14,538,060	17,044,447	9

**Table 4:** Candidate genes for the response to heat stress and UV-light radiation underlying consensus genomic regions in which QTL related to chlorophyll fluorescence or stomatal dynamics co-locate. For each candidate gene, the QTL code of the region where it is located, the Gene ID, the corresponding functional annotation and supporting evidence are reported. Genes predicted in consensus regions with EV > 10% are highlighted in bold. Abbreviations: HS = heat stress, WDS = water deficit stress

QTL code	Gene ID	Annotation	Supporting evidence
*Signal perception, transduction and transcriptional control*			
trQTL_3.3	Vitvi03g00614	Somatic embryogenesis receptor kinase 1	Down-regulated by recovery from heat stress (HS) (Liu et al. 2012)
cfQTL_4.2	Vitvi04g00728	Histidine Kinase 5	Involved in response to different stresses (Pham and Desikan 2012)
trQTL_4.2	Vitvi04g01151	Auxin efflux carrier component 5	Down-regulated by HS (Zha et al. 2020)
**trQTL_5.1**	**Vitvi05g00540**	**Serine/threonine-protein kinase HT1**	**Down-regulated by HS** (Zha et al. 2020)
**trQTL_5.1**	**Vitvi05g00542**	**Protein NETWORKED 1A**	**Down-regulated by HS** (Liu et al. 2012)
trQTL_5.2	Vitvi05g00640	Transcription factor bHLH92	Up-regulated by HS (Rocheta et al. 2014; Liu et al. 2020a); up-regulated by combined HS-WDS (Ju et al. 2021)
**cfQTL_7.1**	**Vitvi07g01408**	**Probable 1-deoxy-D-xylulose-5-phosphate synthase 2, chloroplastic**	**Up-regulated by HS** (Liu et al. 2012)
**cfQTL_7.1**	**Vitvi07g01425**	**Protein ecdysoneless-like**	**Up-regulated by HS** (Rocheta et al. 2014)
**cfQTL_7.1**	**Vitvi07g01457**	**14-3-3-like protein GF14 kappa**	**Up-regulated by HS** (Cheng et al. 2018)
**cfQTL_7.1**	**Vitvi07g01488**	**Homeobox-leucine zipper protein ATHB-6**	**Down-regulated by HS** (Rocheta et al. 2014)
**cfQTL_7.1**	**Vitvi07g01498**	**14-3-3-like protein**	**Down-regulated by HS** (Liu et al. 2012)
**trQTL_9.1**	**Vitvi09g00321**	**Ankyrin repeat-containing protein ITN1-like**	**Down-regulated by HS** (Zha et al. 2020)
**trQTL_9.1**	**Vitvi09g00323**	**Dehydration-responsive element-binding protein 3**	**Down-regulated by HS, WDS and combined HS-WDS** (Rocheta et al. 2014; Ju et al. 2021)
**trQTL_9.1**	**Vitvi09g00355**	**Dynein light chain LC6, flagellar outer arm**	**Down-regulated by HS** (Liu et al. 2012; Zha et al. 2020)
**trQTL_9.1**	**Vitvi09g00393**	**Trihelix transcription factor ASIL1**	**Candidate gene for chlorophyll content** (Tafesse et al. 2020)
cfQTL_10.1	Vitvi10g00398	PAP-specific phosphatase HAL2-like	Down-regulated by HS (Zha et al. 2020)
cfQTL_10.1	Vitvi10g00417	Protein STRUBBELIG-RECEPTOR FAMILY 1-like isoform X2	Up-regulated by HS (Liu et al. 2012; Rocheta et al. 2014)
cfQTL_11.1	Vitvi11g00030	Sucrose synthase	Down-regulated by HS (Liu et al. 2012, 2020a; Rocheta et al. 2014)
cfQTL_11.1	Vitvi11g00045	Ethylene-responsive transcription factor ERF017	Up-regulated by HS, WDS and combined HS-WDS (Liu et al. 2012, 2020a; Rocheta et al. 2014; Zhao et al. 2014)
cfQTL_11.1	Vitvi11g00046	Ethylene-responsive transcription factor ERF016	Up-regulated by HS (Zhao et al. 2014)
**cfQTL_12.1**	**Vitvi12g00181**	**Serine/threonine-protein kinase EDR1**	**Down-regulated by HS** (Rocheta et al. 2014)
cfQTL_13.1	Vitvi13g00852	GDSL esterase/lipase At5g03610	Down-regulated by HS (Zha et al. 2020)
cfQTL_13.2	Vitvi13g01358	Probable leucine-rich repeat receptor-like protein kinase At1g35710	Down-regulated by HS (Zha et al. 2020)
cfQTL_13.2	Vitvi13g01396	65-kDa microtubule-associated protein 5	Up-regulated by HS (Zha et al. 2020)
cfQTL_15.1	Vitvi15g01077	Mitogen-activated protein kinase homolog MMK2	Involved in ROS metabolism (Nakagami et al. 2006)
cfQTL_17.1	Vitvi17g00157	Protein SHOOT GRAVITROPISM 5	Down-regulated by HS (Rocheta et al. 2014)
**cfQTL_19.1, trQTL_19.1**	**Vitvi19g01284**	**C2 domain-containing protein At1g53590**	**Up-regulated by HS** (Rocheta et al. 2014)
**cfQTL_19.1**	**Vitvi19g01356, Vitvi19g04443**	**9-cis-epoxycarotenoid dioxygenase 1**	**Up-regulated during WDS and combined HS-WDS** (Lehr et al. 2022)**; up-regulated by HS** (Guo et al. 2022)
**cfQTL_19.1, trQTL_19.1**	**Vitvi19g02230**	**Gibberellin 2-beta-dioxygenase 1**	**Up-regulated by HS** (Zha et al. 2020; Upadhyay and Upadhyay 2021)
*ROS homeostasis*			
**cfQTL_7.1**	**Vitvi07g01502**	**Peroxidase 17**	**Down-regulated by HS** (Zha et al. 2020)
**trQTL_9.1**	**Vitvi09g00375**	**Pyridoxal 5′-phosphate synthase-like subunit PDX1.2**	**Vitvi09g00375 up-regulated by HS** (Liu et al. 2012; Rocheta et al. 2014; Jiang et al. 2017; Lecourieux et al. 2020; Zha et al. 2020)
	**Vitvi09g04108**		
	**Vitvi09g04109**		
**trQTL_9.1**	**Vitvi09g01604**	**Putative Peroxidase 48**	**Down-regulated by HS** (Upadhyay and Upadhyay 2021)
cfQTL_11.1	Vitvi11g00038	Peroxiredoxin Q	Involved in protecting photosynthesis (Carvalho et al. 2015)
cfQTL_12.2	Vitvi12g01624	Peroxidase 43	Down-regulated by HS (Rocheta et al. 2014)
*Protein homeostasis*			
**cfQTL_4.1**	**Vitvi04g00148**	**Thermospermine synthase ACAULIS5**	**Involved in protection from HS-induced damage** (Sagor et al. 2013)
**trQTL_5.1**	**Vitvi05g00545**	**Stress-induced-phosphoprotein 1**	**Co-chaperone involved in response to stress** (Toribio et al. 2020)
**cfQTL_7.1**	**Vitvi07g01393**	**Chaperone protein Dnaj A6, chloroplastic**	**Up-regulated by HS** (Rocheta et al. 2014; Zhen et al. 2023)**; involved in the response to various stresses, including HS** (Chen et al. 2021)**; playing a positive role in heat stress resistance** (Zhen et al. 2023)
**cfQTL_7.1**	**Vitvi07g01524**	**Heat shock 70 Kda protein, mitochondrial**	**Up-regulated by HS or combined HS-WDS** (Rocheta et al. 2014; Zha et al. 2020; Ju et al. 2021; Guo et al. 2022; Liu et al. 2022)
**cfQTL_7.1**	**Vitvi07g02588**	**Protein CHAPERONE-LIKE PROTEIN OF POR1, chloroplastic**	**Up-regulated by HS** (Zha et al. 2020)
**cfQTL_7.1**	**Vitvi07g03046**	**F-box protein FBW2**	**Down-regulated by HS** (Rocheta et al. 2014)
**trQTL_9.1**	**Vitvi09g00392**	**Heat shock 70 kDa protein 14**	**Up-regulated by HS and combined HS-WDS** (Liu et al. 2014; Rocheta et al. 2014; Zha et al. 2020; Ju et al. 2021; Guo et al. 2022)
cfQTL_10.1	Vitvi10g00370	F-box/LRR-repeat protein 25-like	Vitvi10g01748 up-regulated by HS (Rocheta et al. 2014)
	Vitvi10g01748	F-box protein At1g61340-like	
	Vitvi10g01749	F-box protein At5g03100	
	Vitvi10g01751	Putative F-box protein At1g49610	
cfQTL_11.1	Vitvi11g00044	20 kDa chaperonin, chloroplastic	Up-regulated by HS (Rocheta et al. 2014; Zha et al. 2020)
cfQTL_15.1	Vitvi15g01082	RING-H2 finger protein ATL68	Down-regulated by HS (Upadhyay and Upadhyay 2021)
cfQTL_15.1	Vitvi15g01083,	Late embryogenesis abundant protein At1g64065	Vitvi15g01084 up-regulated by HS (Rocheta et al. 2014)
	Vitvi15g01084*	Late embryogenesis abundant protein Lea14-A	
cfQTL_17.1	Vitvi17g00171	Chaperone protein DnaJ	**–**
**cfQTL_19.1**	**Vitvi19g01339**	**F-box/LRR-repeat protein At4g14103**	**–**
	**Vitvi19g01351**	**F-box/LRR-repeat protein At3g59190**	
	**Vitvi19g02200**	**F-box/LRR-repeat protein At4g14096-like**	
*Osmoprotection*			
**cfQTL_7.1**	**Vitvi07g01389**	**Putative mannitol dehydrogenase**	**Vitvi07g02555 up-regulated by osmotic stress** (Conde et al. 2015) **and by HS** (Lecourieux et al. 2020)
	**Vitvi07g02553**		
	**Vitvi07g02554**		
	**Vitvi07g02555**		
	**Vitvi07g02556**		
**cfQTL_7.1**	**Vitvi07g04623**	**Probable mannitol dehydrogenase**	**Up-regulated by HS** (Rocheta et al. 2014; Lecourieux et al. 2020)
*Water and ion movement*			
cfQTL_3.2	Vitvi03g01288	zinc transporter 8	Down-regulated by HS (Liu et al. 2012)
**trQTL_5.1**	**Vitvi05g00546**	**Sulfate transporter 3.1**	**Up-regulated by HS** (Upadhyay and Upadhyay 2021)
**trQTL_9.1**	**Vitvi09g00350**	**Sulfite exporter TauE/SafE family protein 3**	**Down-regulated by HS** (Rocheta et al. 2014; Zha et al. 2020)
**trQTL_9.1**	**Vitvi09g00399**	**Probable sulfate transporter 3.4**	**Up-regulated by HS** (Rocheta et al. 2014)
cfQTL_10.1	Vitvi10g00385	Syntaxin-124	Up-regulated by HS (Zha et al. 2020)
cfQTL_15.1	Vitvi15g01072	Mechanosensitive ion channel protein 1, mitochondrial	Involved in maintaining redox homeostasis under HS (Lee et al. 2016)
cfQTL_17.1	Vitvi17g00162	Potassium transporter 1-like isoform X1/NF-X1-type zinc finger protein NFXL1	Down-regulated by HS (Liu et al. 2012)
*Photosynthesis and respiration*			
**cfQTL_4.1**	**Vitvi04g00151**	**NAD(P)H-quinone oxidoreductase subunit U, chloroplastic**	**Involved in HS response** (Liu et al. 2014)
**trQTL_9.1**	**Vitvi09g00361**	**Photosystem I reaction center subunit VI, chloroplastic**	**Down-regulated by HS** (Guo et al. 2022)
cfQTL_10.1	Vitvi10g00372	Chlorophyll a-b binding protein 13, chloroplastic	Involved in HS response (Liu et al. 2014)
cfQTL_10.1	Vitvi10g00399	Internal alternative NAD(P)H-ubiquinone oxidoreductase A1, mitochondrial	Involved in HS response (Liu et al. 2014)
cfQTL_10.1	Vitvi10g04257	External alternative NAD(P)H-ubiquinone oxidoreductase B2, mitochondrial	Involved in HS response (Liu et al. 2014)
	Vitvi10g04258		
cfQTL_13.2	Vitvi13g01328	Ribulose bisphosphate carboxylase/oxygenase activase, chloroplastic	Driver of heat tolerance (Kurek et al. 2007; Phillips et al. 2022)
cfQTL_13.2	Vitvi13g02302	Membrane-associated protein VIPP1, chloroplastic	Function in maintaining chloroplast membranes (Zhang and Sakamoto 2015)
*Response to radiation*			
**cfQTL_3.1**	**Vitvi03g01243**	**Protein FAR-RED IMPAIRED RESPONSE 1**	**Involved in the response to UV-radiation and temperature stress** (Dai et al. 2022)
**cfQTL_3.1**	**Vitvi03g01244**	**Protein FAR1-RELATED SEQUENCE 2**	**Involved in the response to UV-radiation and temperature stress** (Dai et al. 2022)
cfQTL_3.2	Vitvi03g01304	Ultraviolet-B Receptor UVR8	Involved in UV-radiation protection (Loyola et al. 2016); up-regulated by HS (Zha et al. 2020)
cfQTL_4.2	Vitvi04g00693	Protein SUPPRESSOR OF PHYA-105 1	Involved in light signaling (Baumgardt et al. 2002)
cfQTL_13.2	Vitvi13g01385	Protein MAINTENANCE OF PSII UNDER HIGH LIGHT 1	Involved in protecting photosystem II from photodamage (Liu and Last 2015)
*Other*			
**trQTL_5.1**	**Vitvi05g00538**	**Pentatricopeptide repeat-containing protein At3g22690**	**Down-regulated by HS** (Upadhyay and Upadhyay 2021)
**cfQTL_7.1**	**Vitvi07g01482**	**Bifunctional riboflavin biosynthesis protein RIBA 1, chloroplastic**	**Candidate gene for photochemical reflective index** (Tafesse et al. 2020)
**trQTL_9.1**	**Vitvi09g01610**	**Pentatricopeptide repeat-containing protein At3g16010**	**Candidate gene for chlorophyll content** (Tafesse et al. 2020)**; up-regulated by HS** (Zha et al. 2020)
cfQTL_10.1	Vitvi10g02336	Polyphenol oxidase, chloroplastic	Possibly involved in abiotic stress responses (Boeckx et al. 2015); Vitvi10g04289 modulated by HS (Liu et al. 2012)
	Vitvi10g04287		
	Vitvi10g04289		
	Vitvi10g04292		
cfQTL_12.2	Vitvi12g01622	Serine carboxypeptidase-like 42	Involved in response to different stresses (Wang et al. 2021a)
cfQTL_12.2	Vitvi12g01636	Pentatricopeptide repeat-containing protein At2g36240	Down-regulated by HS (Zha et al. 2020)
cfQTL_13.1	Vitvi13g04266	GDSL esterase/lipase	Involved in response to different stresses (Ding et al. 2019)

## Discussion

Chlorophyll fluorescence and stomatal dynamics were evaluated in relation to heat stress in a grapevine mapping population grown under field conditions, with the aim of adding evidence to abiotic stress tolerance studies conducted so far on potted plants under semi-controlled conditions (Marguerit et al. 2012; Coupel-Ledru et al. 2014, 2016, 2024; Su et al. 2020; Brault et al. 2021; Trenti et al. 2021). Given the strong influence of environmental conditions on these physiological traits, accurate phenotyping required multiple evaluation sessions conducted over three consecutive years, utilizing an optimized protocol for both measurement and data processing. For QTL analysis, the difference between stress and non-stress conditions was normalized for control values to compensate for observed variations, as reported in a similar study (Talukder et al. 2014). Differences in plant chlorophyll fluorescence between datasets of the same year can be ascribed to differences in phenological stages. Namely, flowering and berry pea-size are the most susceptible stages to changing temperature (Venios et al. 2020). Differences between datasets collected in different years depend, instead, mainly on seasonal environmental conditions and on plants’ vigor. Maximum temperatures experienced by vines in various sampling sessions over the 3 years were comprised between 27.3 and 36.8 °C, with differences between morning and afternoon sessions comprised between 11.5 and 17.9 °C. Those temperatures should not be considered as severe heat stress for grapevine, because photosynthesis starts to be highly affected when temperatures exceed 40 °C. However, at 35 °C, heat may trigger the onset of several acclimation mechanisms within the photosynthetic apparatus (Venios et al. 2020). Therefore, climatic conditions experienced by plants in the field, despite not being high enough for severe stress, were suitable for the study’s purpose.

In this experiment, 16 stable QTL clusters were significantly associated with quantum efficiency of photosystem II (*F*_*v*_*/F*_*m*_) and other chlorophyll fluorescence parameters and explained 5.5–16.3% of the phenotypic variation (Fig. 3, Table 2, Table S 3.1, Online Resource 3). In addition, 10 stable QTL clusters were identified in relation to stomatal dynamics with an explained variance between 8.1 and 12.5% (Fig. 4, Table 3, Table S 3.2, Online Resource 3), one of which corresponds to a QTL cluster for chlorophyll fluorescence (*cfQTL_19.1*).

The identification of several QTL explaining a low percentage of total phenotypic variance for both chlorophyll fluorescence and transpirational parameters highlights the complex genetic architecture of the evaluated traits, similarly to what was reported for major crops.

Candidate genes by position in QTL clusters with explained variance above 10%, for which an involvement in heat stress response in grapevine has been reported (Liu et al. 2012, 2020a; Rocheta et al. 2014, 2016; Jiang et al. 2017; Zha et al. 2020; Ju et al. 2021; Upadhyay and Upadhyay 2021; Guo et al. 2022; Tan et al. 2023), allowed us to outline biological mechanisms implicated in heat stress response relevant in our experiment (Table 4).

### Signal perception, transduction and transcriptional control

The initial stress signals (e.g., variations in membrane fluidity, osmotic or ionic effects, oxidative stress due to ROS (reactive oxygen species) generation, and protein denaturation) trigger downstream signaling cascades and transcriptional controls that activate stress-responsive mechanisms to re-establish homeostasis and protect/repair damaged proteins and membranes. Some broad groups of signal transduction molecules are the calcium-dependent protein kinases (CDPKs), mitogen-activated protein kinases (MAPK/MPKs), sugars (as signaling molecules), phytohormones, as well as nitric oxide (NO) and hydrogen peroxide (H_2_O_2_). Evidence of the activation of these mechanisms was provided by candidate genes within two of the most robust QTL clusters, *cfQTL_12.1* and *cfQTL_19.1.* The gene Vitvi19g01284 codes for a C2 domain-containing protein belonging to the calcium-dependent lipid-binding (CaLB domain) family protein. Calcium signaling has been widely reported to play a crucial role in raising plant thermotolerance (Kang et al. 2024). While the serine/threonine-protein kinase EDR 1 (MAPK) gene (Vitvi12g00181) is known to be involved in the regulation of plant disease resistance but also in abiotic stress responses (Tang et al. 2005), including changing temperatures (Kim et al. 2003). Other protein kinases with supporting evidence from previous grapevine transcriptomic studies (see Table 4) are a somatic embryogenesis receptor kinase (Vitvi03g00614), a serine/threonine-protein kinase HT1 (Vitvi05g00540), a STRUBBELIG-RECEPTOR family member (Vitvi10g00417)*,* a leucine-rich repeat receptor-like protein kinase (Vitvi13g01358), and a kinase-interacting protein (Vitvi05g00542); while Vitvi04g00728 and Vitvi15g01077 code for a histidine kinase 5 and a mitogen-activated kinase, respectively, for which an important role in ROS homeostasis is reported also in response to abiotic stimuli (Nakagami et al. 2006; Pham and Desikan 2012).

All major phytohormones, such as ABA, auxin, brassinosteroids, cytokinins, ethylene, gibberellins, jasmonic acid and salicylic acid, have been reported to play critical roles in plants’ response to heat stress as signaling molecules (Ahammed and Yu 2016). Studies on grapevine under heat stress, drought stress and combined stress, highlighted the involvement of ABA and of the enzyme NCED1 (9-cis-epoxycarotenoid dioxygenase), which catalyzes a very initial step in ABA biosynthetic pathway, in drought and combined stresses (Lehr et al. 2022). Moreover, in the experiment done by (Rocheta et al. 2014), both heat stress and water deficit stress led to moderate up-regulation of some ABA-responsive genes and ABA-related transcription factors, which makes ABA signaling genes important targets for abiotic stress resistance in a broad sense, not just for drought resistance. Based on these findings and on the observation that *OsNCED1* confers heat stress tolerance in rice seedling plants (Zhang et al. 2022), we propose that the genes Vitvi19g01356 and Vitvi19g04443 may also contribute to heat stress response in grapevine. Other genes involved in ABA signaling are a homeobox-leucine zipper protein (Vitvi07g01488) and an ankyrin repeat-containing protein ITN1 (increased tolerance to NaCl1)-like (Vitvi09g00321) that may affect the ABA-mediated production of ROS (Sakamoto et al. 2008). A dehydration-responsive element-binding protein (Vitvi09g00323) as well as two ethylene-responsive transcription factors (Vitvi11g00045 and Vitvi11g00046) identified in this work, all belonging to the AP2/EREBP (APETALA2/ethylene-responsive element binding proteins) family of transcription factors, were reported to exhibit a strong differential expression upon heat stress, drought and combined stress exposure (see Table 4). Therefore, also ethylene seems to have a central role in both heat and drought stress pointing to a stress-mediated cross-talk in the ethylene signaling pathway, evidenced by similar expression of ethylene-responsive genes (Rocheta et al. 2014). Finally, genes coding for an auxin efflux carrier component (Vitvi04g01151), for a protein involved in auxin-mediated signaling (Vitvi07g01425) and for a gibberellin 2-beta-dioxygenase (Vitvi19g02230) support the role of these plant hormones in grapevine response to increasing temperatures. Other genes with a role in signal perception and transduction and with supporting evidence in literature are a dynein light chain LC6 (Vitvi09g00355), a 14–3-3-like protein (Vitvi07g01457 and Vitvi07g01498) and a trihelix transcription factor gene (Vitvi09g00393) (see Table 4).

In addition to the above signal transduction molecules, volatile terpenes have been reported to play a role in relieving oxidative and thermal stresses in plants (Loreto and Schnitzler 2010). In particular, Bertamini et al. showed how leaf monoterpene emission reduced photosynthetic downregulation under natural high temperature conditions in field-grown grapevine. This stress resilience seems to be mediated by the increased ability to counteract the negative effect of ROS either directly via scavenging, or indirectly via thylakoid membrane stabilization. However, the most likely hypothesis is that monoterpenes act as a signaling molecule (Bertamini et al. 2021). Interestingly, in this work we found a gene (Vitvi07g01408) coding for DXS (1-deoxy-D-xylulose-5-phosphate synthase), the first enzyme of the plastidial isoprenoid biosynthetic pathway. This gene is strongly expressed in young leaves and up-regulated by heat stress (see Table 4).

### ROS homeostasis

Evidence of the involvement of mechanisms related to ROS homeostasis was found in different QTL clusters, with genes coding for three peroxidases (Vitvi07g01502, Vitvi09g01604, Vitvi12g01624), which were all reported to be down-regulated under heat stress, together with a peroxiredoxin Q (Vitvi11g00038) involved in protecting photosynthesis (Carvalho et al. 2015). Moreover, three genes coding for the positive regulator of vitamin B_6_ biosynthesis pyridoxal 5′-phosphate synthase PDX1.2 (Vitvi09g00375, Vitvi09g04108 and Vitvi09g04109) were also found, the former of which is strongly up-regulated in different grapevine transcriptomic studies (see Table 4)*.* Vitamin B_6_ has an important role as antioxidant and its increase upon heat stress has been reported in literature (Moccand et al. 2014).

### Protein homeostasis

Genes coding for heat shock proteins (HSPs) were found in the confidence intervals of four consensus genomic regions: two 70 kDa heat shock proteins (Vitvi07g01524 and Vitvi09g00392), a 20 kDa chaperonin (Vitvi11g00044), two chaperone proteins DnaJ (Vitvi07g01393 and Vitvi17g00171) and a chaperone-like (Vitvi07g02588). Heat shock proteins, like HSP70 and low molecular weight proteins, are widely known to be involved in plant heat stress response and acquisition of thermotolerance (Wahid et al. 2007), playing a central role in preventing the misfolding and aggregation of proteins and helping their proper refolding (Venios et al. 2020). They are also engaged in facilitating the proteolytic degradation of unstable proteins by targeting them to lysosomes or proteasomes. Our results are in line with findings from the literature reporting an overexpression of HSPs in grapevine in response to heat stress (Liu et al. 2014). Bernfur et al*.* also demonstrated the association of HSP21 to thylakoid membranes in heat stressed plants, suggesting a role in stabilization of the membrane (Bernfur et al. 2017). DnaJ proteins serve as co-chaperones of HSP70, playing crucial roles in the response to various stresses, including heat stress (Chen et al. 2021). For example, tobacco plants overexpressing *VvDNAJA6* (Vitvi07g01393) and *VvHSP70* showed a high-temperature tolerance phenotype, thanks to the accumulation of ROS-scavenging enzymes and osmotic substances like proline (Zhen et al. 2023). An increasing number of studies suggest indeed that the HSPs/chaperones can play a role in stress signal transduction/gene activation and interact with other stress-response mechanisms such as the production of antioxidants and osmolytes (Wang et al. 2004). Furthermore, we found a stress-induced phosphoprotein 1 (STIP1, Vitvi05g00545), also known as HOP, which is a co-chaperone of HSP70/HSP90 and is involved in plant heat stress response (Toribio et al. 2020). The synthesis of heat-shock proteins can be influenced by polyamines that are known to provide protection to plants from heat stress in different ways (Hasanuzzaman et al. 2013). In line with this, a gene coding for thermospermine synthase (Vitvi04g00148) has a potential role in protection from heat stress-induced damage (Sagor et al. 2013). With respect to protein fate, we also identified some genes implicated in ubiquitination and proteolysis (Vitvi07g03046, Vitvi10g00370, Vitvi10g01748, Vitvi10g01749, Vitvi10g01751, Vitvi15g01082, Vitvi19g01339, Vitvi19g01351 and Vitvi19g02200). Indeed, F-box-like proteins are known to play a major role in plant responses to various developmental and stress conditions, including heat (Guérin et al. 2021).

### Osmoprotection

Six genes coding for mannitol dehydrogenase (Vitvi07g01389, Vitvi07g02553, Vitvi07g02554, Vitvi07g02555, Vitvi07g02556, Vitvi07g04623) were found in *cfQTL_7.1*. Together with amino acids and quaternary amines, polyol and sugars are known to play a role as osmolytes (Wang et al. 2003). Their primary function is to maintain cell turgor, but they can also act as ROS scavengers or chemical chaperones maintaining protein structures and functions. In particular, mannitol has been recognized to play a role in coping with heat stress-induced oxidative damage and excessive solar irradiance (Lecourieux et al. 2020).

### Photosynthesis and respiration

Genes involved in the regulation of the photosynthetic process were found in several stable QTL clusters identified in this study. Among them are genes for a Photosystem I reaction center subunit (Vitvi09g00361), a ribulose bisphosphate carboxylase/oxygenase activase (Rubisco activase, Vitvi13g01328), a membrane-associated protein VIPP1 (Vitvi13g02302), some NAD(P)H-quinone oxidoreductase (Vitvi04g00151; Vitvi10g00399, Vitvi10g04257 and Vitvi10g04258), and a chlorophyll a-b binding protein (Vitvi10g00372). It is already known from the literature that photoinhibition caused by heat stress is related to Rubisco inactivation and to the thermal instability of Rubisco activase, which is involved in the step of Rubisco re-activation (Salvucci and Crafts-Brandner 2004). Moreover, it has been found that an enhanced thermal stability of Rubisco activase can confer higher thermotolerance and an improved photosynthetic activity under heat stress (Kurek et al. 2007; Phillips et al. 2022). VIPP1 is a membrane-associated protein with multiple roles, including the maintenance of the photosynthetic complex in thylakoid membranes (Zhang and Sakamoto 2015). Its involvement has been proposed in relation to membrane stress, such as for example osmotic stress, but heat stress is also known to cause damage to thylakoid membranes by increasing their fluidity, with consequences also for PSII (Wahid et al. 2007; Venios et al. 2020). Hence both Rubisco activase and VIPP1 could play a role in grapevine photosynthetic response to heat stress. Conversely, NAD(P)H-quinone oxidoreductase and chlorophyll a-b binding protein role in grapevine heat stress response is supported by a proteomic study (Liu et al. 2014).

### Response to radiation

Two genes coding for a Far-Red Impaired Response 1 (FAR1, Vitvi03g01243) and a protein FAR1-Related Sequence (FRS, Vitvi03g01244) were found in *cfQTL_3.1*, a genomic region conserved in the 3 years of analysis and with explained variance 8.1–16.3%. FAR1 belongs to the wider FRS protein family and together with Far-Red Elongated Hypocotyls 3 (FHY3) it is known to be responsible for the translocation of phytochrome A in response to far red light (Wang and Wang 2015). Furthermore, FAR1/FHY3 are involved in UV-B signaling, in the circadian rhythm regulation, and in ROS homeostasis (Wang and Wang 2015). Moreover, Dai et al*.* reported their involvement in salt and temperature stress responses (Dai et al. 2022), which suggests a potential role in plant response not only to UV-light radiation stress but also to heat stress. In other QTL regions, additional genes involved in the response to UV light were found coding for an ultraviolet-B receptor UVR8 (Vitvi03g01304), a protein suppressor of Phytochrome A (PhyA) (SPA1, Vitvi04g00693), and a Maintenance of PSII Under High Light 1 (MPH1, Vitvi13g01385). The protein UVR8 has been studied also in grapevine, and its fundamental role in the UV-B signaling pathway was demonstrated, as it regulates a huge number of genes involved in UV-radiation protection (Brown et al. 2005; Loyola et al. 2016). Conversely, SPA1 is known to play a role in the light-signal transduction (Baumgardt et al. 2002), while MPH1 codes for a proline-rich protein that helps the maintenance of normal PSII activity under photoinhibitory stress and protects PSII from photooxidative damages (Liu and Last 2015).

## Conclusions

For perennial fruit crops, this study on grapevine is the first attempt to identify QTL and candidate genes related to heat stress response in the vineyard by analyzing photosynthetic and transpirational phenotypes. Among the biological processes implicated in plant heat tolerance, signal perception and transduction emerge as central mechanisms in our experiment, considering that two of the four most conserved and significant genomic regions harbor genes encoding key signaling components. Notably, these include 9-cis-epoxycarotenoid dioxygenase (NCED) and serine/threonine-protein kinase EDR1. Though part of broad classes of signaling molecules, these proteins have documented roles in plant responses to high temperatures, supporting their potential as functional candidates for heat stress adaptation. Results obtained in this study further support proteostasis, together with osmoprotection, as key adaptive strategies for plant survival under high-temperature conditions. Several genes encoding heat shock proteins (HSPs) and molecular chaperones, in fact, were identified as functional candidates within the most stable genomic regions. These proteins are well-documented for their roles in protecting cellular proteins from heat-induced damage by facilitating proper folding, preventing aggregation, and assisting in refolding or degradation of misfolded proteins. Finally, the identification of genes involved in the maintenance of photosynthetic efficiency and the response to high light and radiation stress supports the hypothesis that preserving photosynthetic function is a critical adaptive response to prolonged heat exposure. These include genes encoding a Rubisco activase, essential for the regeneration of Rubisco activity under thermal stress; NAD(P)H-quinone oxidoreductases, which play a role in chloroplast redox homeostasis and electron transport; Far-Red Impaired Response 1 (FAR1), involved in light signaling and circadian regulation; and Maintenance of PSII Under High Light 1 (MPH1), which supports the stability and repair of photosystem II under high irradiance conditions. In addition to the candidate genes discussed, from this exploratory analysis other gene predictions in the stable QTL clusters emerged, whose role in grapevine adaptation to and recovery capacity from heat stress may be tested. Overall, this work paves the way for future research on grapevine resilience to rising temperatures, providing a robust workflow for the analysis of physiological traits in the field and suggesting thermotolerance candidate genes for advanced investigations.

## Supplementary Information

Below is the link to the electronic supplementary material.

Supplementary file1 (XLSX 314 KB)

Supplementary file2 (XLSX 500 KB)

Supplementary file3 (XLSX 128 KB)

## References

[CR1] Ahammed GJ, Yu JQ (2016) Plant hormones under challenging environmental factors. Springer, Berlin, pp 1–269. 10.1007/978-94-017-7758-2

[CR2] Angira B, Zhang Y, Scheuring CF et al (2022) Construction of a single nucleotide polymorphism linkage map and identification of quantitative trait loci controlling heat tolerance in cowpea, *Vigna unguiculata* (L.) Walp. Mol Genet Genomics 297:1481–1493. 10.1007/s00438-022-01928-9 35933483 10.1007/s00438-022-01928-9

[CR3] Arab MM, Brown PJ, Abdollahi-Arpanahi R et al (2022) Genome-wide association analysis and pathway enrichment provide insights into the genetic basis of photosynthetic responses to drought stress in Persian walnut. Hortic Res 9:uhac124. 10.1093/hr/uhac124 35928405 10.1093/hr/uhac124 PMC9343916

[CR4] Baumgardt RL, Oliverio KA, Casal JJ, Hoecker U (2002) SPA1, a component of phytochrome A signal transduction, regulates the light signaling current. Planta 215:745–753. 10.1007/s00425-002-0801-x 12244439 10.1007/s00425-002-0801-x

[CR5] Bernfur K, Rutsdottir G, Emanuelsson C (2017) The chloroplast-localized small heat shock protein Hsp21 associates with the thylakoid membranes in heat-stressed plants. Protein Sci 26:1773–1784. 10.1002/pro.3213 28608391 10.1002/pro.3213 PMC5563132

[CR6] Bertamini M, Faralli M, Varotto C et al (2021) Leaf monoterpene emission limits photosynthetic downregulation under heat stress in field-grown grapevine. Plants 10:1–17. 10.3390/plants10010181 10.3390/plants10010181 PMC7835969 33478116

[CR7] Boeckx T, Winters AL, Webb KJ, Kingston-Smith AH (2015) Polyphenol oxidase in leaves: is there any significance to the chloroplastic localization? J Exp Bot 66:3571–3579. 10.1093/jxb/erv141 25873687 10.1093/jxb/erv141

[CR8] Brault C, Doligez A, Cunff L et al (2021) Harnessing multivariate, penalized regression methods for genomic prediction and QTL detection of drought-related traits in grapevine. G3: Genes Genomes Genet 11:jkab248. 10.1093/g3journal/jkab248 10.1093/g3journal/jkab248 PMC8496232 34544146

[CR9] Brown BA, Cloix C, Jiang GH et al (2005) A UV-B-specific signaling component orchestrates plant UV protection. Proc Natl Acad Sci USA 102:18225–18230. 10.1073/pnas.0507187102 16330762 10.1073/pnas.0507187102 PMC1312397

[CR10] Cappetta E, Andolfo G, Guadagno A et al (2021) Tomato genomic prediction for good performance under high-temperature and identification of loci involved in thermotolerance response. Hortic Res 8:212. 10.1038/s41438-021-00647-3 34593775 10.1038/s41438-021-00647-3 PMC8484564

[CR11] Carvalho LC, Vidigal P, Amâncio S (2015) Oxidative stress homeostasis in grapevine (*Vitis vinifera* L.). Front Environ Sci 3:1–15. 10.3389/fenvs.2015.00020

[CR12] Chen J, Chopra R, Hayes C et al (2017) Genome-wide association study of developing leaves’ heat tolerance during vegetative growth stages in a sorghum association panel. Plant Genome 10:1–15. 10.3835/plantgenome2016.09.0091 10.3835/plantgenome2016.09.0091 28724078

[CR13] Chen L, Wang Q, Tang M et al (2021) QTL mapping and identification of candidate genes for heat tolerance at the flowering stage in rice. Front Genet 11:621871. 10.3389/fgene.2020.621871 33552136 10.3389/fgene.2020.621871 PMC7862774

[CR14] Cheng C, Wang Y, Chai F et al (2018) Genome-wide identification and characterization of the 14-3-3 family in *Vitis vinifera* L. during berry development and cold- and heat-stress response. BMC Genomics 19:1–14. 10.1186/s12864-018-4955-8 30068289 10.1186/s12864-018-4955-8 PMC6090852

[CR15] Conde A, Regalado A, Rodrigues D et al (2015) Polyols in grape berry: transport and metabolic adjustments as a physiological strategy for water-deficit stress tolerance in grapevine. J Exp Bot 66:889–906. 10.1093/jxb/eru446 25433029 10.1093/jxb/eru446

[CR16] Coupel-Ledru A, Lebon É, Christophe A et al (2014) Genetic variation in a grapevine progeny (*Vitis vinifera* L. cvs GrenachexSyrah) reveals inconsistencies between maintenance of daytime leaf water potential and response of transpiration rate under drought. J Exp Bot 65:6205–6218. 10.1093/jxb/eru228 25381432 10.1093/jxb/eru228 PMC4223985

[CR17] Coupel-Ledru A, Lebon E, Christophe A et al (2016) Reduced night-time transpiration is a relevant breeding target for high water-use efficiency in grapevine. Proc Natl Acad Sci USA 113:8963–8968. 10.1073/pnas.1600826113 27457942 10.1073/pnas.1600826113 PMC4987834

[CR18] Coupel-Ledru A, Lebon E, Goutouly JP et al (2022) Phenotyping for drought tolerance in grapevine populations: the challenge of heterogeneous field conditions. In: Miguel Costa J, Catarino S, Escalona JM, Comuzzo P (eds) Improving sustainable viticulture and winemaking practices. Elsevier, Amsterdam, pp 65–83

[CR19] Coupel-Ledru A, Westgeest AJ, Albasha R et al (2024) Clusters of grapevine genes for a burning world. New Phytol 242:10–18. 10.1111/nph.19540 38320579 10.1111/nph.19540

[CR20] Dai J, Sun J, Peng W et al (2022) FAR1/FHY3 transcription factors positively regulate the salt and temperature stress responses in *Eucalyptus grandis*. Front Plant Sci 13:1–15. 10.3389/fpls.2022.883654 10.3389/fpls.2022.883654 PMC9115564 35599891

[CR21] Delrot S, Grimplet J, Carbonell-Bejerano P et al (2020) Genetic and genomic approaches for adaptation of grapevine to climate change. In: Kole C (ed) Genomic designing of climate-smart fruit crops. Springer, Berlin, pp 157–270

[CR22] Ding LN, Li M, Wang WJ et al (2019) Advances in plant GDSL lipases: from sequences to functional mechanisms. Acta Physiol Plant 41:1–11. 10.1007/s11738-019-2944-4

[CR23] Fahad S, Bajwa AA, Nazir U et al (2017) Crop production under drought and heat stress: plant responses and management options. Front Plant Sci 8:1–16. 10.3389/fpls.2017.01147 28706531 10.3389/fpls.2017.01147 PMC5489704

[CR24] Faralli M, Matthews J, Lawson T (2019) Exploiting natural variation and genetic manipulation of stomatal conductance for crop improvement. Curr Opin Plant Biol 49:1–7. 10.1016/j.pbi.2019.01.003 30851622 10.1016/j.pbi.2019.01.003 PMC6692497

[CR25] Faralli M, Mallucci S, Bignardi A et al (2024) Four decades in the vineyard: the impact of climate change on grapevine phenology and wine quality in northern Italy. OENO One 58:1–21

[CR26] Gallo AE, Perez Peña JE, Prieto JA (2021) Mechanisms underlying photosynthetic acclimation to high temperature are different between *Vitis vinifera* cv. Syrah and Grenache. Funct Plant Biol 48:342–357. 10.1071/FP20212 33278910 10.1071/FP20212

[CR27] Greer DH (2017) Responses of biomass accumulation, photosynthesis and the net carbon budget to high canopy temperatures of *Vitis vinifera* L. cv. Semillon vines grown in field conditions. Environ Exp Bot 138:10–20. 10.1016/j.envexpbot.2017.03.001

[CR28] Greer DH (2019) Short-term temperature dependency of the photosynthetic and PSII photochemical responses to photon flux density of leaves of *Vitis vinifera* cv. Shiraz vines grown in field conditions with and without fruit. Funct Plant Biol 46:634–648. 10.1071/FP18324 30967170 10.1071/FP18324

[CR29] Greer DH, Weedon MM (2012) Modelling photosynthetic responses to temperature of grapevine (*Vitis vinifera* cv. Semillon) leaves on vines grown in a hot climate. Plant Cell Environ 35:1050–1064. 10.1111/j.1365-3040.2011.02471.x 22150771 10.1111/j.1365-3040.2011.02471.x

[CR30] Greer DH, Weston C (2010) Heat stress affects fl owering, berry growth, sugar accumulation and photosynthesis of *Vitis vinifera* cv. Semillon grapevines grown in a controlled environment. Funct Plant Biol 37:206–214

[CR31] Guérin C, Mouzeyar S, Roche J (2021) The landscape of the genomic distribution and the expression of the f-box genes unveil genome plasticity in hexaploid wheat during grain development and in response to heat and drought stress. Int J Mol Sci 22:1–19. 10.3390/ijms22063111 10.3390/ijms22063111 PMC8002965 33803701

[CR32] Guo R, Lin L, Huang G et al (2022) Transcriptome analysis of ‘Kyoho’ grapevine leaves identifies heat response genes involved in the transcriptional regulation of photosynthesis and abscisic acid. Agronomy 12:2591. 10.3390/agronomy12102591

[CR33] Hasanuzzaman M, Nahar K, Alam MdM et al (2013) Physiological, biochemical, and molecular mechanisms of heat stress tolerance in plants. Int J Mol Sci 14:9643–9684. 10.3390/ijms14059643 23644891 10.3390/ijms14059643 PMC3676804

[CR34] Hochberg U, Batushansky A, Degu A et al (2015) Metabolic and physiological responses of Shiraz and Cabernet Sauvignon (*Vitis vinifera* L.) to near optimal temperatures of 25 and 35 °C. Int J Mol Sci 16:24276–24294. 10.3390/ijms161024276 26473851 10.3390/ijms161024276 PMC4632749

[CR35] IPCC (2023) Climate Change 2023: synthesis report. Contribution of working groups I, II and III to the sixth assessment report of the intergovernmental panel on climate change, pp 35–115. 10.59327/IPCC/AR6-9789291691647

[CR36] Jagadish SVK, Way DA, Sharkey TD (2021) Plant heat stress: concepts directing future research. Plant Cell Environ 44:1992–2005. 10.1111/pce.14050 33745205 10.1111/pce.14050

[CR37] Jiang J, Liu X, Liu C et al (2017) Integrating omics and alternative splicing reveals insights into grape response to high temperature. Plant Physiol 173:1502–1518. 10.1104/pp.16.01305 28049741 10.1104/pp.16.01305 PMC5291026

[CR38] Jones GV, Davis RE (2000) Climate influences on grapevine phenology, grape composition, and wine production and quality for Bordeaux, France. Am J Enol Vitic 51:249–261. 10.5344/ajev.2000.51.3.249

[CR39] Ju Y, Min Z, Zhang Y et al (2021) Transcriptome profiling provide new insights into the molecular mechanism of grapevine response to heat, drought, and combined stress. Sci Hortic 286:110076. 10.1016/j.scienta.2021.110076

[CR40] Kadir S (2006) Thermostability of photosynthesis of *Vitis aestivalis* and *V. vinifera*. J Am Soc Hortic Sci 131:476–483. 10.21273/jashs.131.4.476

[CR41] Kang X, Zhao L, Liu X (2024) Calcium signaling and the response to heat shock in crop plants. Int J Mol Sci 25:324. 10.3390/IJMS25010324 10.3390/ijms25010324 PMC10778685 38203495

[CR42] Kilasi NL, Singh J, Vallejos CE et al (2018) Heat stress tolerance in rice (*Oryza sativa* L.): identification of quantitative trait loci and candidate genes for seedling growth under heat stress. Front Plant Sci 871:1578. 10.3389/fpls.2018.01578 10.3389/fpls.2018.01578 PMC6221968 30443261

[CR43] Kim JA, Agrawal GK, Rakwal R et al (2003) Molecular cloning and mRNA expression analysis of a novel rice (*Oryza sativa* L.) MAPK kinase kinase, OsEDR1, an ortholog of *Arabidopsis* AtEDR1, reveal its role in defense/stress signalling pathways and development. Biochem Biophys Res Commun 300:868–876. 10.1016/S0006-291X(02)02944-3 12559953 10.1016/s0006-291x(02)02944-3

[CR44] Kurek I, Thom KC, Bertain SM et al (2007) Enhanced thermostability of *Arabidopsis* rubisco activase improves photosynthesis and growth rates under moderate heat stress. Plant Cell 19:3230–3241. 10.1105/tpc.107.054171 17933901 10.1105/tpc.107.054171 PMC2174701

[CR45] Lecourieux D, Kappel C, Claverol S et al (2020) Proteomic and metabolomic profiling underlines the stage- and time-dependent effects of high temperature on grape berry metabolism. J Integr Plant Biol 62:1132–1158. 10.1111/jipb.12894 31829525 10.1111/jipb.12894

[CR46] Lee CP, Maksaev G, Jensen GS et al (2016) MSL1 is a mechanosensitive ion channel that dissipates mitochondrial membrane potential and maintains redox homeostasis in mitochondria during abiotic stress. Plant J 88:809–825. 10.1111/tpj.13301 27505616 10.1111/tpj.13301 PMC5195915

[CR47] Lehr PP, Hernández-Montes E, Ludwig-Müller J et al (2022) Abscisic acid and proline are not equivalent markers for heat, drought and combined stress in grapevines. Aust J Grape Wine Res 28:119–130. 10.1111/ajgw.12523

[CR48] Liu J, Last RL (2015) MPH1 is a thylakoid membrane protein involved in protecting photosystem II from photodamage in land plants. Plant Signal Behav 10:e1076602. 10.1080/15592324.2015.1076602 26337456 10.1080/15592324.2015.1076602 PMC4883845

[CR49] Liu GT, Wang JF, Cramer G et al (2012) Transcriptomic analysis of grape (*Vitis vinifera* L.) leaves during and after recovery from heat stress. BMC Plant Biol 12:174. 10.1186/1471-2229-12-174 23016701 10.1186/1471-2229-12-174 PMC3497578

[CR50] Liu GT, Ma L, Duan W et al (2014) Differential proteomic analysis of grapevine leaves by iTRAQ reveals responses to heat stress and subsequent recovery. BMC Plant Biol 14:1–17. 10.1186/1471-2229-14-110 10.1186/1471-2229-14-110 PMC4108046 24774513

[CR51] Liu X, Fan Y, Mak M et al (2017) QTLs for stomatal and photosynthetic traits related to salinity tolerance in barley. BMC Genomics 18:1–13. 10.1186/s12864-016-3380-0 28049416 10.1186/s12864-016-3380-0 PMC5210286

[CR52] Liu M, Ju Y, Min Z et al (2020a) Transcriptome analysis of grape leaves reveals insights into response to heat acclimation. Sci Hortic 272:109554. 10.1016/j.scienta.2020.109554

[CR53] Liu Y, Jiang J, Fan X et al (2020b) Identification of heat tolerance in Chinese wildgrape germplasm resources. Horticulturae 6:1–7. 10.3390/horticulturae6040068

[CR54] Liu X, Chen H, Li S, Wang L (2022) Genome-wide identification of the Hsp70 gene family in grape and their expression profile during abiotic stress. Horticulturae 8:743. 10.3390/horticulturae8080743

[CR55] Loreto F, Schnitzler JP (2010) Abiotic stresses and induced BVOCs. Trends Plant Sci 15:154–166. 10.1016/j.tplants.2009.12.006 20133178 10.1016/j.tplants.2009.12.006

[CR56] Loyola R, Herrera D, Mas A et al (2016) The photomorphogenic factors UV-B RECEPTOR 1, ELONGATED HYPOCOTYL 5, and HY5 HOMOLOGUE are part of the UV-B signalling pathway in grapevine and mediate flavonol accumulation in response to the environment. J Exp Bot 67:5429–5445. 10.1093/jxb/erw307 27543604 10.1093/jxb/erw307 PMC5049392

[CR57] Luo HB, Ma L, Xi HF et al (2011) Photosynthetic responses to heat treatments at different temperatures and following recovery in grapevine (*Vitis amurensis* L.) leaves. PLoS ONE 6:1–11. 10.1371/journal.pone.0023033 10.1371/journal.pone.0023033 PMC3162573 21887227

[CR58] Marguerit E, Brendel O, Lebon E et al (2012) Rootstock control of scion transpiration and its acclimation to water deficit are controlled by different genes. New Phytol 194:416–429. 10.1111/j.1469-8137.2012.04059.x 22335501 10.1111/j.1469-8137.2012.04059.x

[CR59] Maxwell K, Johnson GN (2000) Chlorophyll fluorescence—a practical guide. J Exp Bot 51:659–668. 10.1016/j.rsci.2018.02.001 10938857 10.1093/jxb/51.345.659

[CR60] Moccand C, Boycheva S, Surriabre P et al (2014) The pseudoenzyme PDX1.2 boosts vitamin B6 biosynthesis under heat and oxidative stress in *Arabidopsis*. J Biol Chem 289:8203–8216. 10.1074/jbc.M113.540526 24505140 10.1074/jbc.M113.540526 PMC3961649

[CR61] Mohanty JK, Thakro V, Yadav A et al (2024) Delineation of genes for a major QTL governing heat stress tolerance in chickpea. Plant Mol Biol 114:19. 10.1007/s11103-024-01421-4 38363401 10.1007/s11103-024-01421-4

[CR62] Molina-Bravo R, Arellano C, Sosinski BR, Fernandez GE (2011) A protocol to assess heat tolerance in a segregating population of raspberry using chlorophyll fluorescence. Sci Hortic 130:524–530. 10.1016/j.scienta.2011.07.022

[CR63] Monneveux P, Pastenes C, Reynolds MP (2003) Limitations to photosynthesis under light and heat stress in three high-yielding wheat genotypes. J Plant Physiol 160:657–666. 10.1078/0176-1617-00772 12872488 10.1078/0176-1617-00772

[CR64] Murchie EH, Lawson T (2013) Chlorophyll fluorescence analysis: a guide to good practice and understanding some new applications. J Exp Bot 64:3983–3998. 10.1093/jxb/ert208 23913954 10.1093/jxb/ert208

[CR65] Nakagami H, Soukupová H, Schikora A et al (2006) A mitogen-activated protein kinase kinase kinase mediates reactive oxygen species homeostasis in *Arabidopsis*. J Biol Chem 281:38697–38704. 10.1074/jbc.M605293200 17043356 10.1074/jbc.M605293200

[CR66] OIV (International Organisation of Vine and Wine) (2023) State of the world vine and wine sector. OIV Press Conference, pp 168–169. 10.1787/9789264244047-44-en

[CR67] Van Ooijen JW (2009) MapQTL 6.0 software for mapping quantitative trait loci in experimental populations of diploid species

[CR68] Paliwal R, Röder MS, Kumar U et al (2012) QTL mapping of terminal heat tolerance in hexaploid wheat (*T. aestivum* L.). Theor Appl Genet 125:561–575. 10.1007/s00122-012-1853-3 22476874 10.1007/s00122-012-1853-3

[CR69] Palliotti A, Silvestroni O, Petoumenou D (2009) Photosynthetic and photoinhibition behavior of two field-grown grapevine cultivars under multiple summer stresses. Am J Enol Vitic 60:189–198

[CR70] Pham J, Desikan R (2012) Modulation of ROS production and hormone levels by AHK5 during abiotic and biotic stress signaling. Plant Signal Behav 7:893–897. 10.4161/psb.20692 22827948 10.4161/psb.20692 PMC3474678

[CR71] Phillips AL, Scafaro AP, Atwell BJ (2022) Photosynthetic traits of Australian wild rice (*Oryza australiensis*) confer tolerance to extreme daytime temperatures. Plant Mol Biol 110:347–363. 10.1007/s11103-021-01210-3 34997897 10.1007/s11103-021-01210-3 PMC9646608

[CR72] Poudyal D, Rosenqvist E, Ottosen CO (2019) Phenotyping from lab to field-tomato lines screened for heat stress using *F*_*v*_/*F*_*m*_ maintain high fruit yield during thermal stress in the field. Funct Plant Biol 46:44–55. 10.1071/FP17317 10.1071/FP17317 30939257

[CR73] Rocheta M, Becker JD, Coito JL et al (2014) Heat and water stress induce unique transcriptional signatures of heat-shock proteins and transcription factors in grapevine. Funct Integr Genomics 14:135–148. 10.1007/s10142-013-0338-z 24122211 10.1007/s10142-013-0338-z

[CR74] Rocheta M, Coito JL, Ramos MJN et al (2016) Transcriptomic comparison between two *Vitis vinifera* L. varieties (Trincadeira and Touriga Nacional) in abiotic stress conditions. BMC Plant Biol 16:1–19. 10.1186/s12870-016-0911-4 27733112 10.1186/s12870-016-0911-4 PMC5062933

[CR75] Sagor GHM, Berberich T, Takahashi Y et al (2013) The polyamine spermine protects *Arabidopsis* from heat stress-induced damage by increasing expression of heat shock-related genes. Transgenic Res 22:595–605. 10.1007/s11248-012-9666-3 23080295 10.1007/s11248-012-9666-3

[CR76] Sakamoto H, Matsuda O, Iba K (2008) ITN1, a novel gene encoding an ankyrin-repeat protein that affects the ABA-mediated production of reactive oxygen species and is involved in salt-stress tolerance in *Arabidopsis thaliana*. Plant J 56:411–422. 10.1111/j.1365-313X.2008.03614.x 18643991 10.1111/j.1365-313X.2008.03614.x

[CR77] Salvucci ME, Crafts-Brandner SJ (2004) Relationship between the heat tolerance of photosynthesis and the thermal stability of rubisco activase in plants from contrasting thermal environments. Plant Physiol 134:1460–1470. 10.1104/pp.103.038323 15084731 10.1104/pp.103.038323 PMC419822

[CR78] Santos JA, Fraga H, Malheiro AC et al (2020) A review of the potential climate change impacts and adaptation options for European viticulture. Appl Sci (Switz) 10:1–28. 10.3390/app10093092

[CR79] Sharma DK, Andersen SB, Ottosen CO, Rosenqvist E (2012) Phenotyping of wheat cultivars for heat tolerance using chlorophyll a fluorescence. Funct Plant Biol 39:936–947. 10.1071/FP12100 32480843 10.1071/FP12100

[CR80] Sharma DK, Andersen SB, Ottosen C, Rosenqvist E (2015) Wheat cultivars selected for high *F*_*v*_/*F*_*m*_ under heat stress maintain high photosynthesis, total chlorophyll, stomatal conductance, transpiration and dry matter. Physiol Plant 153:284–298. 10.1111/ppl.12245 24962705 10.1111/ppl.12245

[CR81] Sharma V, Gangurde SS, Nayak SN et al (2023) Genetic mapping identified three hotspot genomic regions and candidate genes controlling heat tolerance-related traits in groundnut. Front Plant Sci 14:1182867. 10.3389/fpls.2023.1182867 37287715 10.3389/fpls.2023.1182867 PMC10243373

[CR82] Sheoran S, Gupta M, Kumari S et al (2022) Meta-QTL analysis and candidate genes identification for various abiotic stresses in maize (*Zea mays* L.) and their implications in breeding programs. Mol Breed 42:26. 10.1007/s11032-022-01294-9 37309532 10.1007/s11032-022-01294-9 PMC10248626

[CR83] Sinsawat V, Leipner J, Stamp P, Fracheboud Y (2004) Effect of heat stress on the photosynthetic apparatus in maize (*Zea mays* L.) grown at control or high temperature. Environ Exp Bot 52:123–129. 10.1016/j.envexpbot.2004.01.010

[CR84] Su K, Xing H, Guo Y et al (2020) High-density genetic linkage map construction and cane cold hardiness QTL mapping for *Vitis* based on restriction site-associated DNA sequencing. BMC Genomics 21:1–14. 10.1186/s12864-020-06836-z 10.1186/s12864-020-06836-z PMC7310074 32571215

[CR85] Sun Y, Liu X, Zhai H et al (2016) Responses of photosystem II photochemistry and the alternative oxidase pathway to heat stress in grape leaves. Acta Physiol Plant 38:1–8. 10.1007/s11738-016-2235-2

[CR86] Sun Y, Geng Q, Du Y et al (2017) Induction of cyclic electron flow around photosystem I during heat stress in grape leaves. Plant Sci 256:65–71. 10.1016/j.plantsci.2016.12.004 28167040 10.1016/j.plantsci.2016.12.004

[CR87] Tafesse EG, Gali KK, Reddy Lachagari VB et al (2020) Genome-wide association mapping for heat stress responsive traits in field pea. Int J Mol Sci 21:2043. 10.3390/ijms21062043 32192061 10.3390/ijms21062043 PMC7139655

[CR88] Talukder SK, Babar MA, Vijayalakshmi K et al (2014) Mapping QTL for the traits associated with heat tolerance in wheat (*Triticum aestivum* L.). BMC Genet 15:1–13. 10.1186/s12863-014-0097-4 25384418 10.1186/s12863-014-0097-4 PMC4234900

[CR89] Tan JW, Shinde H, Hu Y et al (2023) Global transcriptome and gene co-expression network analyses reveal regulatory and non-additive effects of drought and heat stress in grapevine. Front Plant Sci. 10.3389/fpls.2023.1096225 36818880 10.3389/fpls.2023.1096225 PMC9932518

[CR90] Tang D, Christiansen KM, Innes RW (2005) Regulation of plant disease resistance, stress responses, cell death, and ethylene signaling in *Arabidopsis* by the EDR1 protein kinase. Plant Physiol 138:1018–1026. 10.1104/pp.105.060400 15894742 10.1104/pp.105.060400 PMC1150416

[CR91] Toribio R, Mangano S, Fernández-Bautista N et al (2020) HOP, a co-chaperone involved in response to stress in plants. Front Plant Sci 11:1–8. 10.3389/fpls.2020.591940 33193548 10.3389/fpls.2020.591940 PMC7658193

[CR92] Trenti M, Lorenzi S, Bianchedi PL et al (2021) Candidate genes and SNPs associated with stomatal conductance under drought stress in *Vitis*. BMC Plant Biol 21:1–21. 10.1186/s12870-020-02739-z 33407127 10.1186/s12870-020-02739-z PMC7789618

[CR93] Upadhyay A, Upadhyay AK (2021) Global transcriptome analysis of heat stress response of grape variety. Vitis - J Grapevine Res 60:143–151. 10.5073/vitis.2021.60.143-151

[CR94] Van Leeuwen C, Destrac-Irvine A, Dubernet M et al (2019) An update on the impact of climate change in viticulture and potential adaptations. Agronomy 9:1–20. 10.3390/agronomy9090514

[CR95] Velt A, Frommer B, Blanc S et al (2023) An improved reference of the grapevine genome reasserts the origin of the PN40024 highly homozygous genotype. G3: Genes Genomes Genet 13:1–14. 10.1093/g3journal/jkad067 10.1093/g3journal/jkad067 PMC10151409 36966465

[CR96] Venios X, Korkas E, Nisiotou A, Banilas G (2020) Grapevine responses to heat stress and global warming. Plants 9:1–15 10.3390/plants9121754 PMC7763569 33322341

[CR97] Vervalle JA, Costantini L, Lorenzi S et al (2022) A high-density integrated map for grapevine based on three mapping populations genotyped by the *Vitis*18K SNP chip. Theor Appl Genet 135:4371–4390. 10.1007/s00122-022-04225-6 36271055 10.1007/s00122-022-04225-6 PMC9734222

[CR98] Wahid A, Gelani S, Ashraf M, Foolad MR (2007) Heat tolerance in plants: an overview. Environ Exp Bot 61:199–223. 10.1016/j.envexpbot.2007.05.011

[CR99] Wang H, Wang H (2015) Multifaceted roles of FHY3 and FAR1 in light signaling and beyond. Trends Plant Sci 20:453–461. 10.1016/j.tplants.2015.04.003 25956482 10.1016/j.tplants.2015.04.003

[CR100] Wang W, Vinocur B, Altman A (2003) Plant responses to drought, salinity and extreme temperatures: towards genetic engineering for stress tolerance. Planta 218:1–14. 10.1007/s00425-003-1105-5 14513379 10.1007/s00425-003-1105-5

[CR101] Wang W, Vinocur B, Shoseyov O, Altman A (2004) Role of plant heat-shock proteins and molecular chaperones in the abiotic stress response. Trends Plant Sci 9:244–252. 10.1016/j.tplants.2004.03.006 15130550 10.1016/j.tplants.2004.03.006

[CR102] Wang H, Zhao S, Mao K et al (2018) Mapping QTLs for water-use efficiency reveals the potential candidate genes involved in regulating the trait in apple under drought stress. BMC Plant Biol 18:1–19. 10.1186/s12870-018-1308-3 29940853 10.1186/s12870-018-1308-3 PMC6019725

[CR103] Wang XC, Wu WM, Zhou BB et al (2021a) Genome-wide analysis of the SCPL gene family in grape (*Vitis vinifera* L.). J Integr Agric 20:2666–2679. 10.1016/S2095-3119(20)63587-0

[CR104] Wang Y, Xin H, Fan P et al (2021b) The genome of Shanputao (*Vitis amurensis*) provides a new insight into cold tolerance of grapevine. Plant J 105:1495–1506. 10.1111/tpj.15127 33300184 10.1111/tpj.15127

[CR105] Willits DH, Peet MM (2001) Measurement of chlorophyll fluorescence as a heat stress indicator in tomato: laboratory and greenhouse comparisons. J Am Soc Hortic Sci 126:188–194. 10.21273/jashs.126.2.188

[CR106] Xiao F, Yang ZQ, Lee KW (2016) Photosynthetic and physiological responses to high temperature in grapevine (*Vitis vinifera* L.) leaves during the seedling stage. J Hortic Sci Biotechnol. 10.1080/14620316.2016.1211493

[CR107] Xu H, Liu G, Liu G et al (2014) Comparison of investigation methods of heat injury in grapevine (*Vitis*) and assessment to heat tolerance in different cultivars and species. BMC Plant Biol 14:1–10 10.1186/1471-2229-14-156 PMC4099030 24898786

[CR108] Yamada M, Hidaka T, Fukamachi H (1996) Heat tolerance in leaves of tropical fruit crops as measured by chlorophyll fluorescence. Sci Hortic 67:39–48. 10.1016/S0304-4238(96)00931-4

[CR109] Yeh CH, Kaplinsky NJ, Hu C, Charng YY (2012) Some like it hot, some like it warm: phenotyping to explore thermotolerance diversity. Plant Sci 195:10–23. 10.1016/j.plantsci.2012.06.004 22920995 10.1016/j.plantsci.2012.06.004 PMC3430125

[CR110] Zha Q, Xi X, Jiang A et al (2016a) Changes in the protective mechanism of photosystem II and molecular regulation in response to high temperature stress in grapevines. Plant Physiol Biochem 101:43–53. 10.1016/j.plaphy.2016.01.024 26852109 10.1016/j.plaphy.2016.01.024

[CR111] Zha Q, Xi X, Jiang A, Tian Y (2016b) High temperature affects photosynthetic and molecular processes in field-cultivated *Vitis vinifera* L. × *Vitis labrusca* L. Photochem Photobiol 92:446–454. 10.1111/php.12584 26946321 10.1111/php.12584

[CR112] Zha Q, Xi X, Jiang A, Tian Y (2018) Comparison of the activities of photosystem II of four table grapevine cultivars during high-temperature stress. Hortic Environ Biotechnol 59:363–371. 10.1007/s13580-018-0041-z

[CR113] Zha Q, Xi X, He Y, Jiang A (2020) Transcriptomic analysis of the leaves of two grapevine cultivars under high-temperature stress. Sci Hortic 265:109265. 10.1016/j.scienta.2020.109265

[CR114] Zhang L, Sakamoto W (2015) Possible function of VIPP1 in maintaining chloroplast membranes. Biochim Biophys Acta Bioenerg 1847:831–837. 10.1016/j.bbabio.2015.02.013 10.1016/j.bbabio.2015.02.013 25725437

[CR115] Zhang Y, Liu X, Su R et al (2022) 9-Cis-epoxycarotenoid dioxygenase 1 confers heat stress tolerance in rice seedling plants. Front Plant Sci 13:1–12. 10.3389/fpls.2022.1092630 10.3389/fpls.2022.1092630 PMC9807918 36605966

[CR116] Zhao T, Xia H, Liu J, Ma F (2014) The gene family of dehydration responsive element-binding transcription factors in grape (*Vitis vinifera*): genome-wide identification and analysis, expression profiles, and involvement in abiotic stress resistance. Mol Biol Rep 41:1577–1590. 10.1007/s11033-013-3004-6 24402876 10.1007/s11033-013-3004-6

[CR117] Zhen Z, Dongying F, Yue S et al (2023) Translational profile of coding and non-coding RNAs revealed by genome wide profiling of ribosome footprints in grapevine. Front Plant Sci 14:1097846. 10.3389/fpls.2023.1097846 36844052 10.3389/fpls.2023.1097846 PMC9944039

[CR118] Zhou R, Yu X, Kjær KH et al (2015) Screening and validation of tomato genotypes under heat stress using *F*_*v*_/*F*_*m*_ to reveal the physiological mechanism of heat tolerance. Environ Exp Bot 118:1–11. 10.1016/j.envexpbot.2015.05.006

